# Social-Class Inequalities in Distance Learning During the COVID-19 Pandemic: Digital Divide, Cultural Mismatch, and Psychological Barriers

**DOI:** 10.5334/irsp.716

**Published:** 2023-04-20

**Authors:** Fabian Müller, Sébastien Goudeau, Nicole M. Stephens, Cristina Aelenei, Rasyid Bo Sanitioso

**Affiliations:** 1 Université Paris Cité, Laboratoire de Psychologie Sociale, Boulogne-Billancourt, France; 2 Centre de Recherches sur la Cognition et l’Apprentissage, CNRS, Université de Poitiers, Poitiers, France; 3 Department of Management and Organizations, Kellogg School of Management, Northwestern University, Evanston, IL, USA

**Keywords:** higher education, social-class, digital divide, cultural mismatch, COVID-19

## Abstract

The COVID-19 pandemic forced universities to move towards distance learning, requiring increased use of digital tools and more independent learning from students. In this context, the present study examined two previously documented barriers that contribute to social-class disparities in universities: the digital divide and the experience of cultural mismatch. *Cultural mismatch* refers to the disconnect between the highly independent cultural norms of universities and the interdependent cultural norms common among working-class students. Our goals are to (1) replicate the findings related to these barriers in a European context (2) provide pandemic-specific data related to these barriers, and (3) examine how the digital divide and cultural mismatch relate to psychological factors and learning behaviors necessary for academic success. Two thousand two hundred and seventy-five students in France answered questions about their digital access/use, self-construal, psychological factors (i.e., sense of belonging, self-efficacy, intentions to drop-out from the university), and learning behaviors (e.g., attending class, asking questions). Results showed that working-class students have less digital access and value interdependence more than their middle/upper-class peers, suggesting they are more likely to experience a cultural mismatch. Structural equation modeling revealed that both the digital divide and the experience of cultural mismatch undermines working-class students’ psychological experience (e.g., belonging), which, in turn, hinders their learning behavior. The distance learning required by the pandemic led to increased needs for digital access and independence, and therefore more negatively affected working-class students, which could fuel and widen the social-class achievement gap.

A university student is attending an online class from home. They listen attentively and participate in the lecture by asking questions, and, later, contribute to the discussion. There are no distractions around them. Simultaneously, a fellow classmate is logging in to the online class from a noisy apartment, with their siblings moving around the space. They must relocate to a quieter corner multiple times during the class and turn off their camera to avoid distracting the class. This student is unable to concentrate and to actively participate in class. They feel out of place, but don’t want to stand out, and in turn, begin doubting their ability to succeed academically.

The above vignette illustrates two divergent distance learning experiences shaped by social-class. In the spring of 2020, the closure of schools and universities was detrimental for more than 1.3 billion learners (UNESCO, 2020). The crisis resulted in less on-campus teaching and more distance learning globally and contributed to the widening of the social-class achievement gap (Betthäuser et al., 2022; Engzell et al., 2021; Goudeau et al., 2021). To explain how university closures and the resulting distance learning could have amplified the social-class achievement gap, scholars have argued that working-class students are likely to experience two specific challenges compared to middle/upper-class students: less digital access (i.e., the digital divide) and lower levels of independence in the independent university setting (i.e., cultural mismatch; Goudeau et al., 2021). We use the term *working-class students* to refer to those whose parents don’t have a three-year university degree, or whose parents are employed as blue-collar workers. We use the term *middle/upper-class students* to refer to those who have at least one parent with either a three-year university degree or a professional occupation that requires advanced education or managerial roles (OECD, 2018).

The goal of this paper is threefold. First, we seek to identify whether these two previously documented barriers (i.e., the digital divide and the experience of cultural mismatch) also occur in a context different from the settings where previous cultural mismatch research has been conducted: the French university context. Second, we seek to provide pandemic-specific data related to these barriers that can be used as a comparison point in future research. Third, we seek to examine the extent to which the digital divide and the experience of cultural mismatch predict the psychological barriers that underlie academic inequalities (i.e., differences in belonging, self-efficacy, and intention to drop-out) and learning behaviors (e.g., attending class, asking questions) necessary for academic success.

## The Digital Divide and Cultural Mismatch

### Digital Divide

Research conducted before the COVID-19 pandemic showed a *digital divide*: working-class students have less access to digital/material equipment than do middle/upper-class students (Robinson et al., 2020). However, this issue may have become even more pronounced during the pandemic because participating in online classes requires that students have access to digital equipment (e.g., computer). Although overall digital access has increased over time (World Bank, 2019), digital disparities in access to equipment (e.g., quality of hardware, software, and internet access) persist (González-Betancor et al., 2021). Furthermore, social-class not only shapes access to digital tools, but also how they are used (Harris et al., 2017). Thus, compared to their middle/upper-class peers, working-class students tend to use digital tools more for leisure activities than for educational purposes (Drabowicz, 2017). Finally, the digital divide may be exacerbated because working-class students are less likely to have a dedicated and quiet space (i.e., material equipment; APA Task Force on Socioeconomic Status, 2007), which is also likely to impair their participation during online classes. To mitigate digital divide in France during closure, universities provided portable computers and 4G cards to students (MESRI, 2021). However, universities did not provide a quiet or dedicated place of study to facilitate participation in online classes.

### Cultural Mismatch

Research conducted before the COVID-19 pandemic showed another key barrier that working-class students face: the *cultural mismatch* between the norms of independence that pervade higher education and the relatively interdependent norms more common among working-class students (Phillips et al., 2020; Stephens, Fryberg, et al., 2012; Stephens, Markus, et al., 2014; Stephens, Townsend, et al., 2012). Indeed, middle/upper-class contexts tend to promote cultural norms of independence, thus fostering an independent self-construal that affords an understanding of the self as separate from others and the social environment (Fryberg & Markus, 2007; Stephens, Fryberg, et al., 2012). In contrast, working-class contexts tend to foster a more interdependent self-construal that affords an understanding of the self as connected to others and part of a community (Stephens et al., 2007). The culture of independence in higher education is thus compatible with middle/upper-class students’ family socialization, but presents a mismatch with the interdependent norms more common in working-class contexts. This cultural mismatch has been shown to have negative consequences on working-class students’ subjective experience and performance: It triggers stress, negative emotions, and a lowered sense of belonging, and decreases academic achievement (Phillips et al., 2020; Stephens, Fryberg, et al., 2012; Stephens, Townsend, et al., 2012).

We suggest that the transition to online classes during the pandemic exacerbates cultural mismatch by both (1) requiring more independent behavior of students and (2) emphasizing students’ interdependence while they are learning at home with their families. First, distance learning required even more independence than on-site classes. Social interactions and group work with fellow students and teachers normally facilitated in on-campus settings are less likely to occur. Thus, students need to work individually more often, exert high self-regulation skills (e.g., setting individual goals), and participate more in online vs. in-person settings (e.g., asking questions, voicing their opinions, and answering teacher’s questions; Goudeau et al., 2021). That means to benefit from online classes, students must demonstrate behaviors that reflect independent cultural norms (Stephens, Markus, et al., 2014; Miller & Sperry, 2012). The requirement that students use digital devices (e.g., cameras or microphones) in online learning could also amplify cultural mismatch by emphasizing independence, as being featured on the camera would require that students stand out from the group and be the focus of attention.

Second, the pandemic may also exacerbate cultural mismatch because these independent behaviors required by online learning occur in students’ relatively interdependent home/family contexts. These contexts likely make salient and reinforce working-class students’ interdependent self-construal, which in turn increases their experience of mismatch. Supporting this suggestion, empirical findings showed that the immediate situation (e.g., being at home) shapes the values students endorse (Aelenei et al., 2017).

### Cultural mismatch and digital divide predict psychological factors and learning behaviors

During ordinary times, social-class predicts the psychological factors sense of belonging, self-efficacy, and intention to drop-out. These factors, in turn, predict academic success and drop-out rates (Bandura et al., 1996; Jury et al., 2017, 2019; Phillips et al., 2020; Wiederkehr et al., 2015). For example, when students experience a cultural mismatch or lack the digital resources necessary for online learning, these experiences should predict psychological factors. That is, students may doubt their sense of belonging to university, feel less self-efficacy, and question whether they have what it takes to succeed in the university (Goudeau et al., 2021; Phillips et al., 2020). These psychological experiences, in turn, should lead students to demonstrate fewer learning behaviors (e.g., attending class, asking questions).

## Overview and hypotheses

The current research has three key goals. First, we examine the relevance of the digital divide and cultural mismatch and replicate previous research in a European context. Until now, cultural mismatch has only been studied in elite universities in the US (e.g., Phillips et al., 2020; Stephens et al., 2012). Second, prior to the pandemic, both digital divide and cultural mismatch have been documented as barriers for working-class students. Although these factors are likely to become even more important during the pandemic, they have not yet been examined in this context. To fill this gap, we document the digital divide and cultural mismatch during the pandemic. In doing so, we provide data that can be used in future post-pandemic comparisons. Third, we examine how the digital divide and cultural mismatch relate to psychological factors, and to learning behaviors necessary for academic success.

We seek to accomplish these goals by examining social-class differences in digital divide and in self-construal. We then test how social-class predicts the psychological factors sense of belonging to university, self-efficacy, and intention to drop-out. Third, as distance learning requires more independent behaviors essential for online learning (e.g., asking questions, working alone) and other facilitative learning behaviors (e.g., attending class, listening/reading carefully; i.e., learning behaviors not necessarily related to independence/interdependence), we examine how social-class predicts these behaviors.

*Hypothesis 1*: Compared to middle/upper-class students, we predict that working-class students will:

- Have less digital/material equipment and use this equipment less frequently for educational purposes (digital divide).
- Have higher interdependent and lower independent self-construals (cultural mismatch).
- Experience lower sense of belonging to the university, lower self-efficacy, and higher intentions to drop-out (psychological factors).
- Exhibit fewer learning behaviors essential for online learning (e.g., attending class, asking questions).

We use structural equation modeling to explore if the digital divide and the experience of cultural mismatch (as measured by self-construal) impact psychological factors (e.g., belonging) that can undermine students’ learning behaviors (e.g., attending class, asking questions).

**Figure 1:**
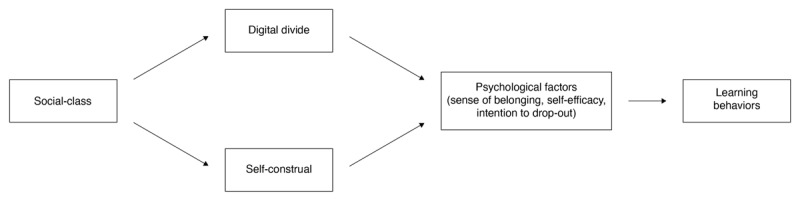
Conceptual model of hypothesis 2.

*Hypothesis 2*: The relationship between social-class and students’ learning behaviors can be explained, in part, by differences in digital divide, self-construal, and psychological factors (Figure 1). We predict that:

- Less digital access and more interdependent and less independent self-construal will predict psychological factors (i.e., lower sense of belonging, lower self-efficacy, and higher intentions to drop-out among working-class students).
- These psychological factors will predict less successful learning behaviors (i.e., attending fewer classes, asking fewer questions).

## Method

This study was preregistered. Study hypotheses, rationale, protocol, variables of interest, sample characteristics, exclusion criteria, analysis strategy (completed on March 29, 2021), data, analysis code, and supplementary materials are openly available at the project’s Open Science Framework page (https://osf.io/qvk4n/?view_only=a264bfc041ff4c889dfff8772b38630e). Data inspection began at the end of data collection. We report all measures, exclusions, and all pre-registered analyses in this study. This study was conducted according to the Ethical Principles of Psychologists and Code of Conduct of the American Psychological Association (2017) as well as the code of conduct of the French Psychology Society (GIRéDeP, 2012). As such, the following ethical guidelines were applied: voluntary participation; anonymized data collection and informed consent, including the possibility of stopping participation at any time. There was no experimental manipulation nor deception and no personal sensitive data was collected, according to the EU’s General Data Protection Regulation (e.g., data relating to religion, politics, health, etc.). Thus, an ethics approval was not required by institutional guidelines or national regulations.

### Operationalizing social-class

Undergraduate students in the social sciences from several universities in France were invited to respond to an online questionnaire shared through professional and social networks during a period of one month (March/April 2021). Applying the exclusion criteria (uncompleted questionnaires = 621, postgraduate students = 19, failing attention checks^1^ = 88) led to a final sample size of 2,275.

Following recent recommendations (Rodríguez-Hernández et al., 2020), we categorized participants’ social-class using two separate proxies: parents’ level of education and parents’ occupations. Details for the classification procedure can be found in the preregistration and in the codebook on the project’s web page. We contrast-coded working-class with -1, and middle/upper-class with 1. Participants who could not be categorized as working-class or middle/upper-class were excluded from analyses,^2^ leading to a final sample of *N_Education_* = 2,170 and of *N_Occupation_* = 1,802 (see Table 1 for participants’ demographics). A sensitivity power analysis (using G*Power software version 3.1.9.4) with the power of 0.80 and α_adjusted_ = .01 (see the Benjamini-Hochberg correction procedure described below) indicated that these two sample sizes allowed us to detect an effect of *f^2^_Education_* = 0.005 (≙ partial eta squared (*η^2^_p, Education_*) = 0.005; *f^2^_Occupation_* = 0.006 ≙ *η^2^_p, Occupation_* = 0.006). Consistent with a conservative effect size (Richard et al., 2003), we considered the sample sizes as reasonable.

**Table 1:** Demographics.


	SOCIAL-CLASS_EDUCATION_		SOCIAL-CLASS_OCCUPATION_	
	
VARIABLES	WORKING-CLASS	MIDDLE/UPPER-CLASS	WORKING-CLASS	MIDDLE/UPPER-CLASS

1. *N*	1247	923	952	850

2. Gender				

Female	86.69%	86.13%	86.24%	86.82%

Male	11.95%	11.38%	12.39%	11.18%

Not specified	0.48%	0.87%	0.53%	0.94%

Self-description	0.88%	1.62%	0.84%	1.06%

3. Year				

First	36.65%	40.30%	37.39%	38.82%

Second	37.77%	34.24%	36.35%	35.53%

Third	25.58%	25.46%	26.26%	25.65%


*Note*: In social-class_Education_, participants were on average 20.37 years old (*SD* = 3.41, *min* = 17, *max* = 56), and the social-class_Occupation_ average was 20.42 years old (*SD* = 3.55, *min* = 17, *max* = 56).

### Measures

#### Digital divide

##### Digital equipment

Participants were asked to report whether or not, at their home, they had access to (1) a desktop computer, (2) a portable computer/laptop/notebook, (3) a tablet, (4) a mobile phone with internet access, (5) internet connection, and (6) high-speed internet. Responses for access to each digital equipment were summed. Participants were also asked to indicate how many of each digital devices they owned (desktop computer, portable computer/laptop/notebook, tablet, and mobile phone) as well as the number of users for each (both from 1 = none to 5 = four or more). Responses were averaged to create composite measures of the number of each digital device and its users.

##### Material equipment

Two items measured the availability of (1) a desk and (2) a quiet place to study at home. Both scales were binary coded (0 = No, 1 = Yes). Responses for each availability were summed.

##### Digital use

Participants were asked how frequently they used the devices (from 1 = almost never to 7 = almost always) for (1) leisure activities, (2) university work, (3) information search related to their studies on social media platforms, and (4) staying in contact with others on social media platforms. Responses were averaged to create composite measures of the frequency of each digital use.

#### Cultural mismatch (measured by self-construal)

We assessed independent and interdependent self-construal with the Motives for Attending College scale (Stephens, Fryberg, et al., 2012). Previous research has shown that independent versus interdependent motives for completing university reflect culture-specific assumptions concerning university education and can be used as an indicator of self-construal. Assuming that university culture in France is seen as independent (Sommet et al., 2015), the endorsement of interdependence indicates cultural mismatch (Phillips et al., 2020). Six items, reflecting interdependent motives for attending college as indicators of interdependent self-construal, represented relationship-oriented reasons (e.g., “I want to bring honor to my family”; α_Education_ and α_Occupation_ = .83). Seven items^3^ reflecting independent motives for attending college as indicators of independent self-construal, represented individual-focused reasons (e.g., “I want to explore new interests”; α_Education_ and α_Occupation_ = .80) for completing university. Items were intermixed. Participants responded using a scale of 1 (not at all important) to 7 (extremely important). Responses were averaged to create composite measures of interdependence and independence.

#### Psychological factors: sense of belonging, self-efficacy, intention to drop-out

Sense of belonging to university was measured with three items (α_Education_ = .74; α_Occupation_ = .73) adapted from two different scales. Two items were taken from Tibbetts et al. (2018; “I belong in [university]” and “I feel like [university] is a good fit for me”) and one item from Trawalter et al. (2020; “I feel ‘out of place’ at [the university]”—reverse coded). Student’s perceived self-efficacy in online classes was measured using five modified items from Midgley et al. (2013) and Stephens, Hamedani, et al. (2014; e.g., “I’m certain I can master the skills taught in online classes this year”; α_Education_ and α_Occupation_ = .90). Students’ intention to drop-out of university was measured using four items from Rump et al. (2017); (1) “I sometimes think about dropping out of university”, (2) “Sometimes I feel unsure if I want to continue my studies”, (3) “It is very unlikely that I will drop out of university”—reverse coded, and (4) “If I had a good alternative, I would drop out of university”; α_Education_ and α_Occupation_ = .76; α_Education_ and α_Occupation_ after dropping item (3) = .84. Items on each scale were intermixed. Participants indicated agreement from 1 (strongly disagree) to 7 (strongly agree).

#### Multi-group confirmatory factor analyses

As we further compared two different student populations (working-class and middle/upper-class), it was necessary to demonstrate that they construed the psychological concepts in the same way. Therefore, we tested different levels of measurement invariance by running a multi-group confirmatory factor analysis (MG-CFA) on the self-construal and psychological variables. Following the guidelines outlined in Gana & Broc (2019), in stage one we conducted a confirmatory factor analysis (CFA) on the overall sample with the software program R (Version 4.0.3) using the package “lavaan” (v0.6-8; Rosseel, 2012). Three indices indicated that our model fit the data well: robust root mean square error of approximation (robust-RMSEA) = .05, 90% confidence interval (CI) [.05, .05]; robust comparative fit index (robust-CFI) = .94; robust Tucker-Lewis index (robust-TLI) = .93 (parameter estimates, Table S3 in Supplemental Material, SM).^4^ Thus, items do saturate on the factors independence, interdependence, sense of belonging, self-efficacy, and intention to drop-out.^5^ In stage two, we tested the CFA model in each group separately. The CFA model’s plausibility for each group was confirmed: for the working-class group, robust-RMSEA = .05, 90% CI [.04, .05], robust-CFI = .95, robust-TLI = .94; for the middle/upper-class group, robust-RMSEA = .05, 90% CI [.05, .06], robust-CFI = .93, robust-TLI = .92. In the following steps, we assessed configural invariance (ensuring that the two groups share the same number of factors and the same factorial pattern), followed by the test of metric invariance (which assumes intergroup equality of factor loadings), and finally the assessment of scalar invariance (assuming intergroup equivalence for both factor loadings and items’ intercepts). Changes in CFI < .01 and in RMSEA < .01 were considered indicators of invariance (Rutkowski & Svetina, 2014). Satisfying these steps would allow us to conclude on a measurement invariance.

The configural invariance was tolerated by the data, robust-RMSEA = .049, 90% CI [.047, .052], robust-CFI = .939, robust-TLI = .930, so we proceeded with the test of metric invariance, robust-RMSEA = .049, 90% CI [.046, .051], robust-CFI = .938, robust-TLI = .932. Comparing these two models confirmed that metric invariance was achieved. Finally, we tested for scalar invariance, with constrained loadings and intercepts, robust-RMSEA = .049, 90% CI [.046, .052], robust-CFI = .935, robust-TLI = .931, and compared it with the metric model, thus confirming that we reached full scalar invariance. Reaching this stage of measurement invariance ensures that if we further document a difference between the two groups, it implies a real difference at the level of the constructs and not a difference that can be imputed to the way they are measured.

#### Learning behaviors

Participants answered questions on the following learning behaviors during a typical week of the pandemic-induced university closure.

##### Class attendance

Six items measured the frequency (1 = almost never to 7 = almost always) for (1) attending online classes, (2) being late for online classes, and (3) missing entire online/distanced classes, as well as (4) attending on-campus class, (5) being late for on-campus classes, and (6) missing entire on-campus classes, when they were offered. Responses were averaged to create composite measures of the frequency of attending, being late, or missing online or on-campus classes.

##### Out-of-class behaviors

Four items measured the frequency (1 = almost never to 7 = almost always) of (1) doing homework and other assignments alone, (2) doing homework and other assignments with fellow students online, (3) looking over class notes, and (4) keeping up with the readings, outside of online classes. Items were intermixed. Responses were averaged to create composite measures for each out-of-class behavior.

##### Independent and other in-class behaviors

Seven items assessed the frequency (1 = almost never to 7 = almost always) of (1) asking questions, (2) participating in discussions, (3) answering questions, (4) switching on their camera, (5) activities non-related to online classes, (6) taking notes, and (7) listening/reading carefully, during online classes. The first four items could be categorized into independent behaviors that match university’s expectations, whereas the last three items are essential for learning but not categorizable as independent or interdependent. Items were intermixed. Responses were averaged to create composite measures for each in-class behavior.

#### Demographics and general information

Year at university was self-reported (1 = first-year; 2 = second-year; 3 = third-year). As a categorical variable, it was coded into two dummy variables with first-year as the reference category. Participants indicated their gender identity as female, male, or they had the option to self-describe. Associations between gender with academic performance and self-construal (Barone & Assirelli, 2020; Markus & Kitayama, 2010) can be seen as a result of access to power and resources (more prevalent among males) that directly affects students’ lives. Thus, we contrast-coded males with further categories (i.e., female and other self-descriptions = –1, male = 1; for analyses we coded gender as numeric in 1 = other categories and 2 = male).

Precise information of all measures can be found in the codebook on the project’s web page.

## Results

### Hypotheses 1: Replication of previous findings in a pandemic-specific online environment

#### Analysis strategy

Table 2 presents means of variables based on social-class, and Table 3 their correlations. Analyses were carried out using the software program R (Version 4.0.3). We present results of robust linear regressions (due to normality and heteroscedasticity issues) with heteroskedasticity-consistent standard error estimators HC4 using the package “sandwich” (v3.0-1; Zeileis, 2004; Zeileis et al., 2020). For categorical data we used binomial logistic models, using the package “robustbase” (v0.93-8; Maechler et al., 2021). We calculated three models, where each dependent variable was regressed on 1) social-class, year, and gender, and 2) on an interactive effect of social-class × year, and social-class × gender, and finally 3) on social-class. Research indicates associations between year and social-class (Phillips et al., 2020), as well as gender with academic performance and self-construal (Barone & Assirelli, 2020; Markus & Kitayama, 2010). To isolate the effects of social-class, we control for year and gender. Overall, the results persist without year and gender as covariates. To maximize power, we controlled the false discovery rate with the Benjamini-Hochberg (BH) procedure (Benjamini & Hochberg, 1995), as it is appropriate to identify effects in large sets (Cramer et al., 2016). First, we ordered the *p*-values resulting from our analyses in an ascending order. Then we computed an adjusted α-level: we multiplied .05 with the division of the rank number of the largest *p*-value divided by the number of analyses. Finally, we compared each *p*-value with the corresponding α_adjusted_. Only those analyses for which the *p*-values fell below the BH threshold were considered to meet the significance criteria, i.e., α_adjusted_ = .01 (see project’s web page). After applying this correction, no interactive effects were found, indicating constant effects of social-class across year and gender. Thus, we present results of the first model, robust to the BH correction. Results for *N_Education_* and *N_Occupation_* did not differ, indicating that both participants’ parents’ level of education and parental occupations are equally good proxies for social-class. For an overview of all results, and consistent with the literature on cultural mismatch that typically focuses on first-generation students (Stephens, Fryberg, et al., 2012), we present results for *N_Education_*. See Table S4 in the supplemental materials.

**Table 2:** Variables depending on social-class_Education_ (*N* = 2170), social-class_Occupation_ (*N* = 1802).


	SOCIAL-CLASS_EDUCATION_					SOCIAL-CLASS_OCCUPATION_				
	
VARIABLES	WORKING-CLASS	MIDDLE/UPPER-CLASS	*T(DF)*	*P*	COHEN’S *D* [95% *CI*]	WORKING-CLASS	MIDDLE/UPPER-CLASS	*T(DF)*	*P*	COHEN’S *D*[95% CI]

**1. Digital Equipment**										

Desktop computer										

- Access	405 (32.5%)	308 (33.4%)	–0.44 (1978.93)	.663	–0.02 [–0.10; 0.07]	305 (32.0%)	291 (34.2%)	–0.99 (1769.99)	.323	–0.05 [–0.14; 0.05]

- Number	1.53 (0.83)	1.57 (0.91)	–0.94 (1886.51)	.347	–0.04 [–0.13; 0.04]	1.53 (0.83)	1.59 (0.90)	–1.53 (1731.33)	.126	–0.07 [–0.17; 0.02]

- Users	1.66 (1.12)	1.69 (1.17)	–0.56 (1933.98)	.579	–0.02 [–0.11; 0.06]	1.68 (1.14)	1.72 (1.20)	–0.72 (1752.73)	.470	–0.03 [–0.13; 0.06]

- Number/Users	1.06 (0.55)	1.08 (0.61)	–0.78 (1866.31)	.433	–0.03 [–0.11; 0.05]	1.06 (0.55)	1.08 (0.62)	–0.77 (1499.10)	.444	–0.04 [–0.13; 0.06]

Portable computer										

- Access	1221 (97.9%)	910 (98.6%)	–1.21 (2142.74)	.228	–0.02 [–0.10; 0.07]	932 (97.9%)	838 (98.6%)	–1.12 (1788.24)	.264	–0.05 [–0.14; 0.04]

- Number	2.69 (1.00)	2.90 (1.11)	–4.58 (1871.43)	<.001***	–0.20 [–0.28; 0.12]	2.67 (0.99)	2.88 (1.11)	–4.31 (1711.12)	<.001***	–0.20 [–0.30; –0.11]

- Users	2.12 (0.98)	2.06 (0.95)	1.60 (2021.95)	.109	0.07 [–0.02; 0.15]	2.14 (0.97)	2.05 (0.97)	1.80 (1774.95)	.072 ^t^	0.09 [–0.01; 0.18]

- Number/Users	1.53 (1.00)	1.75 (1.21)	–4.39 (1763.68)	<.001***	–0.20 [–0.28; 0.11]	1.50 (0.98)	1.75 (1.21)	–4.62 (1413.13)	<.001***	–0.23 [–0.33; –0.14]

Tablet computer										

- Access	423 (33.9%)	340 (36.8%)	–1.40 (1965.83)	.161	–0.06 [–0.15; 0.02]	315 (33.1%)	318 (37.4%)	–1.92 (1764.71)	.055 ^t^	–0.09 [–0.18; 0.00]

- Number	1.54 (0.81)	1.56 (0.83)	–0.67 (1973.59)	.505	–0.03 [–0.11; 0.06]	1.50 (0.78)	1.59 (0.85)	–2.30 (1731.29)	.021*	–0.11 [–0.20; –0.02]

- Users	1.46 (0.86)	1.55 (0.98)	–2.28 (1832.25)	.023*	–0.10 [–0.19; –0.02]	1.47 (0.87)	1.55 (0.97)	–1.83 (1717.64)	.068 ^t^	–0.09 [–0.18; 0.01]

- Number/Users	1.14 (0.58)	1.11 (0.55)	1.21 (2041.07)	0.226	0.05 [–0.03; 0.14]	1.14 (0.58)	1.11 (0.54)	0.90 (1654.25)	.044*	0.04 [–0.05; 0.14]

Mobile phone										

- Access	1237 (99.2%)	913 (98.9%)	0.66 (1809.13)	.507	0.03 [–0.06; 0.11]	944 (99.2%)	841 (98.9%)	0.48 (1712.12)	.634	0.02 [–0.07; 0.12]

- Number	3.24 (1.29)	3.25 (1.32)	–0.16 (1960.02)	.876	–0.01 [–0.09; 0.08]	3.24 (1.29)	3.28 (1.33)	–0.62 (1762.89)	.537	–0.03 [–0.12; 0.06]

- Users	2.05 (1.13)	1.97 (1.09)	1.63 (2030.58)	.104	0.07 [–0.02; 0.16]	2.03 (1.13)	2.01 (1.13)	0.39 (1775.79)	.698	0.02 [–0.07; 0.11]

- Number/Users	2.04 (1.42)	2.11 (1.47)	–1.21 (1950.68)	.225	–0.05 [–0.14; 0.03]	2.05 (1.43)	2.12 (1.48)	–1.06 (1580.97)	.288	–0.05 [–0.15; 0.04]

Access to IC	1225 (98.2%)	907 (98.3%)	–0.05 (1995.80)	.957	–0.00 [–0.09; 0.08]	938 (98.5%)	831 (97.8%)	1.19 (1638.91)	.232	0.06 [–0.04; 0.15]

Access to HSI	690 (55.3%)	529 (57.3%)	–0.92 (1991.99)	.358	–0.04 [–0.13; 0.05]	543 (57.0%)	486 (57.2%)	–0.06 (1777.26)	.953	–0.00 [–0.10; 0.09]

**2. Material equipment**										

Desk to study	1157 (92.8%)	877 (95.0%)	–2.18 (2132.94)	.029*	–0.09 [–0.18; –0.01]	882 (92.6%)	810 (95.3%)	–2.37 (1784.02)	.018*	–0.11 [–0.20; –0.02]

Quiet place to study	998 (80.0%)	783 (84.8%)	–2.93 (2089.50)	.003**	–0.13 [–0.21; –0.04]	744 (78.2%)	736 (86.6%)	–4.74 (1788.94)	<.001***	–0.22 [–0.31; –0.13]

**3. Digital use**										

*f* leisure activities	5.21 (1.58)	5.30 (1.55)	–1.31 (2011.21)	.190	–0.06 [–0.14; 0.03]	5.25 (1.58)	5.28 (1.56)	–0.37 (1781.47)	.711	–0.02 [–0.11; 0.08]

*f* university work	6.32 (1.04)	6.40 (0.99)	–1.93 (2038.48)	.054 ^t^	–0.08 [–0.17; 0.00]	6.30 (1.09)	6.38 (1.03)	–1.71 (1794.65)	.088 ^t^	–0.08 [–0.17; 0.01]

*f* information search	4.91 (1.78)	4.93 (1.81)	–0.23 (1966.74)	.815	–0.01 [–0.10; 0.07]	4.94 (1.77)	4.96 (1.81)	–0.21 (1766.88)	.836	–0.01 [–0.10; 0.08]

*f* staying in contact	5.41 (1.63)	5.50 (1.57)	–1.31 (2027.76)	.190	–0.01 [–0.10; 0.07]	5.40 (1.61)	5.56 (1.50)	–2.09 (1797.11)	.037*	–0.10 [–0.19; –0.01]

**4. Self-construal**										

Independence	5.61 (0.85)	5.73 (0.78)	–3.23 (2067.19)	.001**	–0.14 [–0.22; –0.05]	5.62 (0.83)	5.75 (0.77)	–3.13 (1799.94)	.002**	–0.15 [–0.24; –0.05]

Interdependence	4.60 (1.35)	4.14 (1.36)	7.90 (1976.60)	<.001***	0.34 [0.26; 0.43]	4.67 (1.32)	4.19 (1.35)	7.60 (1762.85)	<.001***	0.36 [0.27; 0.45]

**5. Psychological factors**										

Sense of belonging	4.56 (1.33)	4.63 (1.22)	–1.31 (2069.31)	.191	–0.06 [–0.14; 0.03]	4.57 (1.30)	4.66 (1.21)	–1.23 (1780.75)	.221	–0.06 [–0.15; 0.03]

Self-efficacy	4.56 (1.34)	4.61 (1.36)	–0.76 (1972.52)	.448	–0.03 [–0.12; 0.05]	4.55 (1.35)	4.64 (1.38)	–1.59 (1758.40)	.113	–0.08 [–0.17; 0.02]

Intention to drop-out	3.83 (1.83)	3.50 (1.71)	4.31 (2056.43)	<.001***	0.19 [0.10; 0.27]	3.86 (1.83)	3.43 (1.72)	6.08 (1792.80)	<.001***	0.29 [0.19; 0.38]

**6. Class attendance**										

Attending online class	5.89 (1.68)	5.92 (1.64)	–0.43 (2007.05)	.669	–0.02 [–0.10; 0.07]	5.82 (1.74)	5.91 (1.71)	–1.16 (1783.31)	.246	–0.05 [–0.15; 0.04]

Being late for online class	2.37 (1.67)	2.41 (1.68)	–0.57 (1977.11)	.572	–0.02 [–0.11; 0.06]	2.41 (1.69)	2.36 (1.65)	0.61 (1786.87)	.541	0.03 [–0.06; 0.12]

Missing online class	2.68 (1.77)	2.67 (1.70)	0.05 (2030.15)	.960	0.00 [–0.08; 0.09]	2.69 (1.77)	2.64 (1.70)	0.64 (1790.24)	.525	0.03 [–0.06; 0.12]

Attending on-campus class	2.44 (1.93)	2.54 (1.98)	–1.15 (1957.70)	.249	–0.05 [–0.14; 0.03]	2.45 (1.97)	2.49 (1.96)	–0.46 (1779.36)	.643	–0.02 [–0.11; 0.07]

Being late for on-campus class	1.62 (1.31)	1.74 (1.41)	–2.00 (1900.97)	.045*	–0.09 [–0.17; –0.00]	1.65 (1.36)	1.76 (1.43)	–1.62 (1753.68)	.105	–0.08 [–0.17; 0.02]

Missing on-campus class	2.21 (1.98)	2.11 (1.95)	1.12 (2004.30)	.261	0.05 [–0.04; 0.13]	2.27 (2.07)	2.10 (1.91)	1.75 (1798.30)	.080 ^t^	0.08 [–0.01; 0.17]

**7. Out-of-class behaviors**										

Individual homework	4.76 (1.79)	4.87 (1.87)	–1.29 (1938.48)	.197	–0.06 [–0.14; 0.03]	4.78 (1.83)	4.88 (1.84)	–1.14 (1776.81)	.256	–0.05 [–0.15; 0.04]

Group homework	3.00 (1.89)	2.92 (1.91)	0.99 (1974.71)	.323	0.04 [–0.04; 0.13]	2.96 (1.89)	2.98 (1.92)	–0.30 (1769.96)	.767	–0.01 [–0.11; 0.08]

Looking over class notes	3.39 (1.73)	3.30 (1.76)	1.20 (1967.91)	.231	0.05 [–0.03; 0.14]	3.38 (1.74)	3.35 (1.74)	0.43 (1776.88)	.668	0.02 [–0.07; 0.11]

Keeping up on readings	3.15 (1.75)	3.17 (1.76)	–0.24 (1978.61)	.808	–0.01 [–0.10; 0.07]	3.18 (1.75)	3.19 (1.78)	–0.17 (1769.34)	.864	–0.01 [–0.10; 0.08]

**8. Independent and other in-class behaviors**										

Asking questions	2.70 (1.69)	2.90 (1.70)	–2.70 (1980.12)	.007**	–0.12 [–0.20; –0.03]	2.71 (1.67)	2.93 (1.75)	–2.66 (1757.22)	.007**	–0.13 [–0.22; –0.03]

Participating in discussions	2.99 (1.78)	3.08 (1.78)	–1.23 (1982.74)	.220	–0.05 [–0.14; 0.03]	2.93 (1.75)	3.14 (1.81)	–2.45 (1763.16)	.015*	–0.12 [–0.21; –0.02]

Answering questions	3.28 (1.78)	3.48 (1.75)	–2.66 (2002.40)	.008**	–0.12 [–0.20; –0.03]	3.25 (1.76)	3.51 (1.78)	–3.15 (1772.08)	.002**	–0.15 [–0.24; –0.06]

Switching camera on	1.94 (1.46)	2.21 (1.58)	–4.11 (1897.94)	<.001***	–0.18 [–0.27; –0.10]	1.91 (1.41)	2.30 (1.65)	–5.26 (1680.03)	<.001***	–0.25 [–0.34; –0.16]

Non-related activities	4.27 (1.67)	4.36 (1.63)	–1.23 (2010.86)	.219	–0.05 [–0.14; 0.03]	4.27 (1.66)	4.36 (1.62)	–1.18 (1786.41)	.240	–0.06 [–0.15; 0.04]

Taking notes	4.99 (1.41)	4.93 (1.41)	–0.31 (1996.25)	.757	–0.01 [–0.10; 0.07]	5.00 (1.44)	4.94 (1.43)	0.17 (1787.14)	.861	0.01 [–0.08; 0.10]

Listening/reading carefully	5.73 (1.70)	5.76 (1.68)	1.03 (1989.26)	.302	0.04 [–0.04; 0.13]	5.76 (1.73)	5.74 (1.69)	0.97 (1778.65)	.334	0.05 [–0.05; 0.14]


*Note. N_yes_* (Percentages) for categorical variables, Means (Standard Deviations) for continuous variables. Statistical differences (using Welch’s t-test, Delacre et al., 2017) within variables are highlighted as follows: ^t^ *p* < .10; * *p* < .05; ** *p* < .01; *** *p* < .001. CI = confidence interval, IC = internet connection, HSI = high-speed internet, *f* = frequency.

**Table 3:** Correlations with confidence intervals for social-class_Education_ (*N* = 2170) and social-class_Occupation_ (*N* = 1802).


VARIABLE	1		2		3		4		5		6		7	

1. Social-class														

2. Independence	EDU	.07**												

		[.03, .11]												

	OCC	.07**												

		[.03, .12]												

3. Interdependence	EDU	–.17**	EDU	.21**										

		[–.21, –.13]		[.17, .25]										

	OCC	–.18**	OCC	.21**										

		[–.22, –.13]		[.17, .26]										

4. Sense of belonging	EDU	.03	EDU	.17**	EDU	.05*								

		[–.01, .07]		[.12, .21]		[.01, .09]								

	OCC	.03	OCC	.16**	OCC	.03								

		[–.02, .07]		[.11, .20]		[–.01, .08]								

5. Self-efficacy	EDU	.02	EDU	.12**	EDU	.00	EDU	.29**						

		[–.03, .06]		[.08, .16]		[–.04, .04]		[.25, .33]						

	OCC	.04	OCC	.10**	OCC	.00	OCC	.28**						

		[–.01, .08]		[.05, .14]		[–.04, .05]		[.23, .32]						

6. Intention to drop-out	EDU	–.09**	EDU	–.19**	EDU	.01	EDU	–.43**	EDU	–.41**				

		[–.13, –.05]		[–.23, –.15]		[–.03, .05]		[–.47, –.40]		[–.44, –.37]				

	OCC	–.14**	OCC	–.17**	OCC	.03	OCC	–.43**	OCC	–.41**				

		[–.19, –.10]		[–.21, –.12]		[–.02, .07]		[–.47, –.39]		[–.45, –.37]				

7. Gender	EDU	–.01	EDU	–.03	EDU	–.03	EDU	–.02	EDU	–.01	EDU	.01		

		[–.05, .03]		[–.07, .01]		[–.07, .02]		[–.06, .02]		[–.05, .03]		[–.04, .05]		

	OCC	–.02	OCC	–.04	OCC	–.02	OCC	–.02	OCC	–.01	OCC	.01		

		[–.06, .03]		[–.08, .01]		[–.07, .02]		[–.06, .03]		[–.06, .03]		[–.03, .06]		

8. Year	EDU	–.02	EDU	–.03	EDU	–.03	EDU	–.04	EDU	–.07**	EDU	.08**	EDU	.01

		[–.07, .02]		[–.07, .02]		[–.07, .01]		[–.08, .00]		[–.11, –.03]		[.04, .12]		[–.03, .05]

	OCC	–.01	OCC	–.01	OCC	–.03	OCC	–.05*	OCC	–.06*	OCC	.07**	OCC	.00

		[–.06, .03]		[–.06, .03]		[–.07, .02]		[–.09, –.00]		[–.10, –.01]		[.03, .12]		[–.04, .05]


*Note*: EDU and OCC are used to represent the samples for *N_Education_* and *N_Occupation_*, respectively. Values in square brackets indicate the 95% confidence interval for each correlation. * indicates *p* < .05. ** indicates *p* < .01.

#### Digital divide

##### Digital equipment

We divided the number of owned digital devices by the number of its users to represent real digital access for the digital devices. Working-class students had fewer portable computers/laptops/notebooks than middle/upper-class students, *B* = 0.10, 95% CI [0.06; 0.15], *SE* = 0.02, *t(2165)* = 4.28, *p* < .001, *η^2^_p_* = 0.009. There were no social-class differences for desktop or tablet computers and mobile phones, nor for internet or high-speed internet access (Table S4, SM).

##### Material equipment

Fewer working-class students had a quiet place to study in their home, log-odds = 0.16, 95% CI [0.05; 0.28], *SE* = 0.06, *z(2165)* = 2.80, *p* = .005, *OR* = 1.18, 95% CI [1.05; 1.32], in comparison to their middle/upper-class peers. However, considering our adjusted α-level of .01, there were no social-class differences for access to a desk at which to study, log-odds = 0.19, 95% CI [0.01; 0.38], *SE* = 0.09, *z(2165)* = 2.06, *p* = .039, *OR* = 1.21, 95% CI [1.01; 1.46].

##### Digital use

There were no social-class differences in digital use for leisure activities, university work, information search, or staying in contact with others (Table S4, SM).

#### Cultural mismatch (measured by self-construal)

Middle/upper-class students had more independent self-construal compared to working-class students, *B* = 0.06, 95% CI [0.02; 0.09], *SE* = 0.02, *t(2165)* = 3.17, *p* = .002, *η^2^_p_* = 0.005. In contrast, working-class students endorsed higher interdependent self-construal than middle/upper-class students, *B* = –0.23, 95% CI [–0.29; –0.18], *SE* = 0.03, *t(2165)* = –7.94, *p* < .001, *η^2^_p_* = 0.029.

#### Psychological factors: sense of belonging, self-efficacy, intention to drop-out

Working-class students expressed more intentions to drop-out than did middle/upper-class students, *B* = –0.16, 95% CI [–0.24; –0.09], *SE* = 0.04, *t(2165)* = –4.23, *p* < .001, *η^2^_p_* = 0.008. However, there were no social-class differences for sense of belonging, *B* = 0.03, 95% CI [–0.02; 0.09], *SE* = 0.03, *t(2165)* = 1.22, *p* = .222, *η^2^_p_* = 0.001, or for perceived self-efficacy, *B* = 0.02, 95% CI [–0.04; 0.07], *SE* = 0.03, *t(2165)* = 0.59, *p* = .554, *η^2^_p_* = 0.000.

#### Learning behaviors

##### Class attendance

There were no social-class differences in the frequency of attending, being late, or missing online or on-campus classes (Table S4, SM).

##### Out-of-class behaviors

There were no social-class differences in the frequency of doing homework alone, with fellow students, looking over class notes, or keeping up with the readings when not attending online classes (Table S4, SM).

##### Independent and other in-class behaviors

Working-class students asked fewer questions, *B* = 0.10, 95% CI [0.03; 0.17], *SE* = 0.04, *t(2165)* = 2.68, *p* = .007, *η^2^_p_* = 0.003, and answered questions less frequently, *B* = 0.10, 95% CI [0.02; 0.17], *SE* = 0.04, *t(2165)* = 2.60, *p* = .009, *η^2^_p_* = 0.003, than did middle/upper-class students. Further, middle/upper-class students switched their camera on more often than working-class students, *B* = 0.13, 95% CI [0.07; 0.20], *SE* = 0.03, *t(2165)* = 4.03, *p* < .001, *η^2^_p_* = 0.008. No social-class differences were found for participating in discussions, taking notes, listening/reading carefully, or non-related activities to online classes (Table S4, SM).

### Hypothesis 2: Structural equation model of digital divide, cultural mismatch, psychological factors and learning behaviors

#### Analysis strategy

The recommended minimum sample size for a minimum absolute anticipated effect size of 0.10, with power of 0.80, α = .05, 12 latent variables and 43 observed variables, would be 2,129 (Soper, 2021). Thus, the sample size for *N_Education_* is appropriate.

We fitted structural equation modeling with maximum likelihood estimation with robust (Huber-White) standard errors using the package “lavaan” (v0.6-8; Rosseel, 2012). No system missings occurred. Social-class as categorical variable was contrast-coded as described. First, we calculated a higher-order measurement model with digital divide as the higher-order factor composed of the following lower-order factors: internet access, digital access, material equipment (all three measured with categorical variables), and digital use. The inclusion of these categorical variables led to convergence problems; thus, we defined digital divide as real digital access, i.e., controlled by the number of its users. Accordingly, our final model consisted of lower-order measurement models with six latent factors: digital divide, independent and interdependent self-construal, sense of belonging, self-efficacy, and intention to drop-out, and one higher-order measurement model (i.e., learning behaviors) with five lower-order factors: class attendance online and on-campus, out-of-class behaviors, independent and other in-class behaviors. The following variables with small factor loadings (<.40) were excluded from the low factor models: desktop computers, homework with fellow students, class attendance for on-campus classes.

#### Structural equation model

We hypothesized that social-class differences in digital divide and cultural mismatch would predict differences in psychological factors (sense of belonging, self-efficacy, intentions to drop-out). In turn, these differences in students’ psychological factors would predict differences in learning behaviors (e.g., attending class, asking questions). Three indices indicated that our model fit the data well: RMSEA = .04, 95% CI [.04, .04]; CFI = .90; TLI = .90.

Resulting path coefficients showed that social-class was positively associated with real digital access (β = 0.07, 95% CI = [0.02; 0.13], *SE* = 0.03, *p* = .006). Middle/upper-class students had more real digital access than working-class students. Considering our adjusted α-level of .01, having more real access was not associated with sense of belonging (β = 0.06, 95% CI = [–0.00; 0.11], *SE* = 0.03, *p* = .063), but positively associated with perceived self-efficacy (β = 0.08, 95% CI = [0.02; 0.14], *SE* = 0.03, *p* = .007), and negatively with the intentions to drop-out (β = –0.08, 95% CI = [–0.14; –0.02], *SE* = 0.03, *p* = .006).

Social-class was positively associated with independent self-construal (β = 0.07, 95% CI = [0.03; 0.12], *SE* = 0.02, *p* = .001). Middle/upper-class students endorsed more independent self-construal than did working-class students. The endorsement of independent self-construal was positively associated with sense of belonging (β = 0.24, 95% CI = [0.18; 0.30], *SE* = 0.03, *p* < .001), and with perceived self-efficacy (β = 0.18, 95% CI = [0.12; 0.23], *SE* = 0.03, *p* < .001), but negatively with the intentions to drop-out (β = –0.25, 95% CI = [–0.31; –0.20], *SE* = 0.03, *p* < .001). Those who endorsed independent self-construal more also reported higher sense of belonging and perceived self-efficacy, and less intentions to drop-out.

In addition, social-class was negatively associated with interdependent self-construal (β = –0.18, 95% CI = [–0.22; –0.13], *SE* = 0.02, *p* < .001). Working-class (vs. middle/upper-class) students endorsed more interdependent self-construal. The endorsement of interdependent self-construal was not associated with sense of belonging (β = 0.02, 95% CI = [–0.04; 0.08], *SE* = 0.03, *p* = .462), or with perceived self-efficacy (β = –0.02, 95% CI = [–0.07; 0.04], *SE* = 0.03, *p* = .533), or with the intentions to drop-out (β = 0.04, 95% CI = [–0.02; 0.10], *SE* = 0.03, *p* = .147). Thus, contrary to our hypothesis, those who endorsed interdependent self-construal more did not report lower sense of belonging, lower self-efficacy, or higher intentions to drop-out.

**Figure 2:**
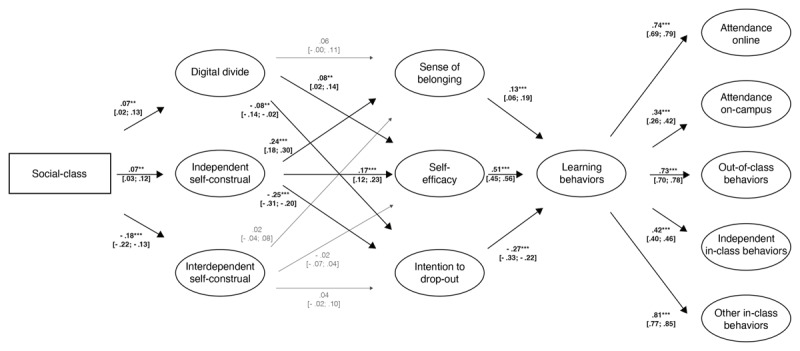
Structural equation model with standardized path coefficients and their confidence intervals in brackets. Significant statistical associations are highlighted in bold and indicated as follows: ** *p* < .01; *** *p* < .001.

Furthermore, sense of belonging (β = 0.13, 95% CI = [0.06; 0.19], *SE* = 0.03, *p* < .001) and perceived self-efficacy (β = 0.51, 95% CI = [0.45; 0.56], *SE* = 0.03, *p* < .001) were both positively associated with students’ learning behaviors, whereas the intentions to drop-out were negatively associated with students’ learning behaviors (β = –0.27, 95% CI = [–0.33; –0.22], *SE* = 0.03, *p* < .001). Those who reported higher sense of belonging and self-efficacy showed better learning behaviors, while those who reported more intentions to drop-out showed worse learning behaviors (see Figure 2).^6^

## Discussion

Pandemic-induced university closures and the resulting move towards distance learning were a challenge for both universities and students. In this study, we examined two factors that could affect working-class students’ psychological experience and their adaptation to online classes: digital divide and cultural mismatch. Our results showed that minimizing digital divide alone (providing equipment for successful class participation such as laptops) is not sufficient. Cultural mismatch was also present during the pandemic. In fact, the pandemic may have exacerbated the cultural mismatch, given that online learning demands more independence and may therefore undermine the learning and performance of working-class students. Moreover, social-class gaps in sense of belonging, self-efficacy, and the intention to drop-out are likely to continue to fuel the social-class achievement gap.

Concerning the digital divide, French universities lend portable computers to students in need during university closures (MESRI, 2021). This should have reduced the digital divide, though we still found social-class differences in access to portable computers. Importantly, working-class students had less access to material resources necessary for optimal distance learning such as a dedicated and quiet place to study and to attend online classes (APA Task Force on Socioeconomic Status, 2007). Indeed, the lack of a dedicated and quiet place may impede students from turning on the cameras and microphones that facilitate active participation in online classes (e.g., asking/answering questions). Thus, this digital divide was linked to independent learning behaviors, necessary in distance learning. Having fewer resources (portable computers, quiet place) could make it particularly difficult for working-class students to adapt successfully to online classes during the lockdown.

As in past studies, we found that working-class (vs. middle/upper-class) students endorsed more interdependent (vs. independent) self-construal (Stephens, Fryberg, et al., 2012). This study generalizes previous findings to other contexts beyond the US, notably to France. We also extend previous findings to a new, online learning context enforced by the global pandemic. We argued that online learning requires even more independence, which may penalize working-class students more than on-site classes. Addtionally, the home setting during online classes likely primes interdependence for working-class students. This cultural mismatch was reflected in students’ learning behaviors. Although working-class students were just as likely to attend online classes (and on-campus classes when they took place), or to do their homework, they were less likely to ask and answer questions during online classes and to switch on their cameras compared to their middle/upper-class peers. Thus, in line with Stephens, Fryberg, et al.’s (2012) findings, our results show that they are less likely to adopt independent behaviors that are important for academic achievement.

Finally, consistent with past findings prior to the pandemic, working-class (vs. middle/upper-class) students stated more intentions to drop-out of a university (Jury et al., 2017). However, contrary to expectations, self-efficacy and sense of belonging to a university did not differ across social-class. This could indicate that the pandemic and the resulting distance learning decreased sense of belonging and self-efficacy among middle/upper-class students. It is also worth pondering if the sense of belonging to a home environment among working-class students might have affected their sense of belonging at a university.

In summary, this research highlights that students experienced distance learning during the pandemic in drastically different ways. Distance learning placed working-class students at even greater digital, material and cultural disadvantage, with significant implications for their learning behaviors and psychological experiences. Having more access to digital devices was associated with psychological factors (high self-efficacy and low intention to drop-out) that are essential for learning behaviors. Thus, though universities supplied computers to decrease the digital divide across social-classes, it remains a factor in academic success, in particular related to the conditions of access to digital equipment (i.e., a quiet room). The results regarding self-construal suggest new insights. The endorsement of independence was positively related to sense of belonging and self-efficacy, and negatively to the intention to drop-out, in line with our predictions. However, contrary to our expectation, interdependence was not related to these psychological factors. These results could have occurred by chance, or may highlight the uniqueness of this cohort compared to pre-pandemic cohorts. As the pandemic-induced distance learning settings required students to work mainly independently, interdependence may not have been a factor in this specific online learning environment. Further, students’ learning behavior was positively associated with the sense of belonging to university and self-efficacy, and negatively with the intention to drop-out. Thus, our results suggest that the endorsement of interdependent self-construal is unrelated to psychological factors that could be essential for online learning. By contrast, the endorsement of independent self-construal is associated with psychological factors (high sense of belonging to university, high self-efficacy, and low intention to drop-out) that facilitate essential learning behaviors.

### Generalizability, limitations, and future directions

Our study provides data of one country (i.e., France), which is a generalization from past studies conducted exclusively in the US. Further, it was conducted in a specific context (i.e., pandemic), that on one hand could explain some unexpected observations (e.g., no link between interdependence and psychological factors, or no difference between self-efficacy and sense of belonging across social-classes), but on the other emphasizes the robustness of factors that could explain social-class differences. That is, these factors remain important and even become more so in the specific context of the pandemic. It thus highlights the importance of providing pandemic-specific data. Pandemic-related university closures happened worldwide, and general similarity in the educational and cultural context can be observed in the so-called Global North (especially between France, Western European and North American countries; Muthukrishna et al., 2020). Thus, our findings support the generalizability of the previous findings to these countries (i.e., WEIRD nations).

The present research has limitations that should be addressed in future work. Though we recruited participants throughout France, our sample mostly included students in social sciences, who may not be representative of all the student population. In addition, most of the participants self-identified as female (>86% of working-class as well as middle/upper-class students). Although we did not find main effects of gender or interactive effects of gender and social-class on self-construal, this bias could have influenced the above-mentioned unexpected observations since past research has shown gender variations in self-construal (Markus & Kitayama, 2010). Future studies should therefore include more diverse and gender-balanced student samples. Other designs, such as longitudinal studies, could also further confirm our findings.

Even though universities tried to reduce digital disparities (e.g., by providing digital equipment or early reopening of libraries), these policies only partially address the structural psychological challenges experienced by working-class students and identified in this study. To fully address these challenges, universities should set up policies inspired by interventions that have proven to be effective. For instance, past research has identified interventions that target students’ self-efficacy and sense of belonging to university: highlighting interdependent working methods (e.g., teamwork; Tibbetts et al., 2018), emphasizing the influence of students’ background on university experiences (i.e., difference-education; Stephens, Hamedani, et al., 2014), and gearing towards continuous assessment (i.e., emphasizing learning- vs. performance-oriented goals; Smeding et al., 2013). Future studies should evaluate empirically their applicability and effectiveness on working-class students’ learning in online settings.

## Conclusion

Our research not only replicated previous findings in the US in a European context during the pandemic, it also showed how the experience of cultural mismatch relates to students’ learning behaviors. Although our study does not examine differences between pandemic and ordinary times, our results are consistent with existing data, showing that the COVID-19 pandemic exacerbated educational inequality across social-classes (Betthäuser et al., 2022). During university closures, both digital divide and cultural mismatch persisted, and the pandemic-driven distance learning in universities may have amplified the resulting social-class achievement gap. Digital disparities paired with pre-existing cultural mismatch could affect the psychological factors that impact students’ learning in crisis-driven distance learning settings.

## Data Accessibility Statement

Preregistration, data, analysis code, and additional online materials are openly available at the project’s Open Science Framework page (https://osf.io/qvk4n/?view_only=a264bfc041ff4c889dfff8772b38630e).

## Additional File

The additional file for this article can be found as follows:

10.5334/irsp.716.s1 Supplemental Online Material. Additional analyses (internal consistency; alternative model and indirect effects for hypothesis 2), and tables S1–S4 (reliability statistics, fit indices, coefficients, regression results).

## Footnotes

Participants had to complete two attentional control questions, asking them to rate their attentiveness to the survey and select a predetermined response from the response options. See codebook on the project’s web page.

In social-class_Education_, participants were considered as working-class if neither parent had a three-year university degree, and as middle/upper-class if at least one parent had a three-year degree. 105 participants did not indicate parents’ level of education. Social-class_Occupation_ was measured by the International Standard Classification of Occupations (ISCO-08; International Labour Organization, 2012). The first two categories were assigned to middle/upper-class (managers/professionals), and the other categories were assigned to working-class (e.g., blue-collar workers, unemployed persons). The third category “Technicians and Associate Professionals” (386 participants) was excluded as middle-class, and the tenth category “Armed Forces Occupations” (41 participants) could not be categorized. 64 participants did not indicate parents’ occupations.

One item has been added to the original scale (see Table S1 in SM for item statistics).

We report indices for *N_Education_*. Similar indices for *N_Occupation_* can be found in Table S2 in SM.

Due to its low factor loading in the CFA (β < 0.30) and an increase in α in the reliability analysis without item (3) of the intention to drop-out scale, it was excluded from further analyses.

See SM for exploratory analyses on indirect effects and an alternative model with an equally good fit.
