# Supplementary material for: Social-Class Inequalities in Distance Learning During the COVID-19 Pandemic: Digital Divide, Cultural Mismatch, and Psychological Barriers
Source: Int Rev Soc Psychol. 2023 Apr 20;36:3. doi: 10.5334/irsp.716 (PMC12372660; doi:10.5334/irsp.716)
Supplement: Supplemental Online Material. — Additional analyses (internal consistency; alternative model and indirect effects for hypothesis 2), and tables S1–S4 (reliability statistics, fit indices, coefficients, regression results). [file irsp-36-716-s1.pdf]

Supplemental Online Material for:

Social-Class Inequalities in Distance Learning during the COVID-19 pandemic: Digital Divide, Cultural Mismatch, and Psychological Barriers

Fabian Müller[1], Sébastien Goudeau[2], Nicole M. Stephens[3], Cristina Aelenei[1], Rasyid Bo Sanitioso[1]

[1] Université Paris Cité, Laboratoire de Psychologie Sociale, Boulogne-Billancourt, France

[2] Centre de Recherches sur la Cognition et l'Apprentissage, CNRS, Université de Poitiers, Poitiers, France

[3] Department of Management and Organizations, Kellogg School of Management, Northwestern University, Evanston, IL, USA

Table of contents

Internal consistency analyses of independent self-construal 3

Hypothesis 2: Structural equation model of digital divide, cultural mismatch, psychological factors and learning behaviors 3

Indirect effects 3

Alternative model 4

References 6

List of Tables

Table S1 *Item reliability statistics for social-class$_{Education}$ (N = 2170) and social-class$_{Occupation}$ (N = 1802)* 7

Table S2 *Summary of fit indices of multi-group confirmatory factor analyses (MG-CFA) for social-class$_{Education}$ (N = 2170) and social-class$_{Occupation}$ (N = 1802)* 9

Table S3 *Standardized and Unstandardized Coefficients for social-class$_{Education}$ (N = 2170) and social-class$_{Occupation}$ (N = 1802)* 10

Table S4 *Results of (robust) regression analyses for social-class$_{Education}$ (N = 2170) and social-class$_{Occupation}$ (N = 1802)* 11

We present here additional analyses and details, that, along with the methodological file, enrich the content in the main text.

Internal consistency analyses of independent self-construal

We assessed independent self-construal with the Motives for Attending College scale (Stephens, Fryberg, et al., 2012). Cronbach's alphas for the original six items ranged from .76 to .80 (Table S1). We added the item "I want to express and communicate my views and thoughts to others", to refer to a tendency often evoked for independence that was not yet represented in the scale. The item statistic ($\alpha_{Education}$ and $\alpha_{Occupation}$ = .80) indicates that this item adequately measures the concept and contributes to the total scale $\alpha_{Education}$ and $\alpha_{Occupation}$ = .80.

Hypothesis 2: Structural equation model of digital divide, cultural mismatch, psychological factors and learning behaviors

We hypothesized that social-class differences in digital divide and cultural mismatch would predict differences in psychological factors (i.e., sense of belonging, self-efficacy, intentions to drop-out). In turn, these differences in students' psychological factors would predict differences in learning behaviors (e.g., attending class, asking questions). We fitted structural equation modelling with maximum likelihood estimation with robust (Huber-White) standard errors using the package "lavaan" (v0.6-8; Rosseel, 2012). No system missings occurred. Social-class as categorical variable was contrast-coded as described. Our final model consisted of lower-order measurement models with six latent factors: digital divide, independent and interdependent self-construal, sense of belonging, self-efficacy, and intention to drop-out, and one higher-order measurement model (i.e., learning behaviors) with five lower-order factors: class attendance online and on-campus, out-of-class behaviors, independent and other in-class behaviors. Three indices indicated that our model fit the data well: RMSEA = .04, 95% CI [.04, .04]; CFI = .90; TLI = .90.

Indirect effects

Exploratory, we tested nine indirect effects of social-class on the psychological factors (sense of belonging, self-efficacy, intention to drop-out) via digital divide and independent as well as interdependent self-construal. Social-class was associated with sense of belonging only via students' independent self-construal (β = 0.02, 95% CI = [0.01; 0.03], SE = 0.01, p = .006), and self-efficacy (β = 0.01, 95% CI = [0.00; 0.03], SE = 0.01, p = .008), while there was no overall effect of social-class on sense of belonging (β = 0.02, 95% CI = [-0.03; 0.08], SE = 0.03, p = .404), and on self-efficacy (β = -0.01, 95% CI = [-0.07; 0.05], SE = 0.03, p = .799). Further, social-class was also via students' independent self-construal indirectly associated with students' intentions to drop out (β = -0.02, 95% CI = [-0.05; -0.01], SE = 0.01, p = .005). Noteworthy is that there was a total effect of students' social-class on intention to drop-out (β = -0.07, 95% CI = [-0.21; -0.05], SE = 0.04, p = .002).

Although sense of belonging and self-efficacy did not differ across social-class, social-class was via students' independent (but not interdependent) self-construal indirectly associated with these psychological factors. Students' independent self-construal could therefore function as a mediator resulting in higher sense of belonging and higher self-efficacy among more independent students who are more likely to be middle/upper-class, which is in line with previous literature (Phillips et al., 2020).

Alternative model

We conducted an exploratory analysis for an alternative model, with the lower-order measurement models with six latent factors: digital divide, independent and interdependent self-construal, sense of belonging, self-efficacy, and intention to drop-out, and one higher-order measurement model (i.e., learning behaviors) with five lower-order factors: class attendance online and on-campus, out-of-class behaviors, independent and other in-class behaviors. As higher drop-out rates can result from psychological barriers such as negative self-efficacy (Jury et al., 2017), in our alternative model the latent factor intention to drop-out is defined as a consequence of the latent factors sense of belonging and self-efficacy. Three indices indicated

that our alternative model fit the data equally well (RMSEA = .04, 95% CI [.04, .04]; CFI = .91; TLI = .90; ΔCFI = 0.006) but did not provide a more meaningful fit (ΔCFI ≥ .01; Chen, 2007).

References

Chen, F. F. (2007). Sensitivity of goodness of fit indexes to lack of measurement invariance. *Structural Equation Modeling: A Multidisciplinary Journal, 14*(3), 464–504. https://doi.org/10.1080/10705510701301834

Jury, M., Smeding, A., Stephens, N. M., Nelson, J. E., Aelenei, C., & Darnon, C. (2017). The experience of low-SES students in higher education: Psychological barriers to success and interventions to reduce social-class inequality. *Journal of Social Issues, 73*(1), 23–41. https://doi.org/10.1111/josi.12202

Phillips, L. T., Stephens, N. M., Townsend, S. S. M., & Goudeau, S. (2020). Access is not enough: Cultural mismatch persists to limit first-generation students' opportunities for achievement throughout college. *Journal of Personality and Social Psychology, 119*(5), 1112–1131. https://doi.org/10.1037/pspi0000234

Rosseel, Y. (2012). lavaan: An R package for structural equation modeling. *Journal of Statistical Software*, *48*(1), 1–36. https://doi.org/10.18637/jss.v048.i02

Stephens, N. M., Fryberg, S. A., Markus, H. R., Johnson, C. S., & Covarrubias, R. (2012). Unseen disadvantage: How American universities' focus on independence undermines the academic performance of first-generation college students. *Journal of Personality and Social Psychology, 102*(6), 1178–1197. https://doi.org/10.1037/a0027143

Table S1

Item reliability statistics for social-class$_{Education}$ (N = 2170) and social-class$_{Occupation}$ (N = 1802)

		Social-class$_{Education}$			Social-class$_{Occupation}$		
Variables	Item content	*M*	*SD*	α	*M*	*SD*	α
Independence							
	I want to explore new interests.	5.58	1.19	0.76	5.59	1.16	0.77
	I want to expand my knowledge of the world.	6.12	0.99	0.76	6.11	1.02	0.76
	I want to become an independent thinker.	5.46	1.47	0.79	5.49	1.44	0.80
	I want to explore my potential in many domains.	5.52	1.23	0.77	5.52	1.23	0.78
	I want to learn more about my interests.	5.72	1.15	0.77	5.73	1.14	0.78
	I want to expand my understanding of the world.	6.11	1.01	0.76	6.12	0.99	0.76
	I want to express and communicate my views and thoughts to other.	5.12	1.43	0.80	5.14	1.41	0.80
Interdependence							
	I want to help my family out after I'm done with college.	4.62	1.83	0.80	4.67	1.81	0.80
	I want to be a role model for people in my community.	4.09	1.78	0.81	4.16	1.76	0.81
	I want to bring honor to my family.	4.23	1.90	0.79	4.29	1.89	0.78
	I want to show that people with my background can do well.	4.05	1.92	0.81	4.11	1.91	0.81
	I want to give back to my community.	4.54	1.75	0.80	4.61	1.73	0.80
	I want to provide a better life for my own children.	4.89	4.89	0.82	4.96	1.93	0.82
Sense of belonging							
	I feel like I belong in college.	4.00	1.57	0.64	4.01	1.54	0.62
	I feel like college is a good fit for me.	4.84	1.49	0.57	4.87	1.47	0.56
	I feel 'out of place' at my university.	4.92	1.68	0.75	4.92	1.68	0.75
Self-efficacy							

		Social-class$_{Education}$			Social-class$_{Occupation}$		
Variables	Item content	*M*	*SD*	α	*M*	*SD*	α
	I'm certain I can master the skills taught in online classes this year.	4.09	1.70	0.88	4.10	1.71	0.88
	I'm certain I can figure out how to do the most difficult work from online classes.	4.27	1.63	0.87	4.26	1.64	0.87
	I can do almost all the work in online class if I don't give up.	4.99	1.59	0.88	5.02	1.58	0.88
	Even if the work from online class is hard, I can learn it.	5.23	1.39	0.88	5.24	1.40	0.88
	I can do even the hardest work in online classes if I try.	4.33	1.70	0.87	4.35	1.72	0.87
Intention to drop-out							
	I sometimes think about dropping out of university.	3.57	2.03	0.61	3.58	2.05	0.61
	Sometimes I feel unsure if I want to continue my studies.	3.88	2.08	0.62	3.90	2.08	0.62
	It is very unlikely that I will drop out of university.	3.43	2.10	0.84	3.39	2.10	0.84
	If I had a good alternative, I would drop out of university.	3.62	2.05	0.68	3.59	2.05	0.68

Note. Means, Standard Deviations, and Cronbach alphas for each item.

Table S2

Summary of fit indices of multi-group confirmatory factor analyses (MG-CFA) for social-class$_{Education}$ (N = 2170) and social-class$_{Occupation}$ (N = 1802)

	Social-class$_{Education}$					Social-class$_{Occupation}$				
	RMSEA	[95% *CI*]		CFI	TLI	RMSEA	[95% *CI*]		CFI	TLI
**Stage 1**										
CFA$_{Overall}$	0.049	[0.046;	0.052]	0.940	0.931	0.048	[0.046;	0.051]	0.941	0.933
**Stage 2**										
CFA$_{Working\text{-}class}$	0.047	[0.044;	0.051]	0.945	0.937	0.050	[0.046;	0.054]	0.938	0.930
CFA$_{Upper\text{-}middle\text{-}class}$	0.052	[0.048;	0.056]	0.930	0.921	0.047	[0.042;	0.051]	0.943	0.935
Configurial invariance	0.049	[0.047;	0.052]	0.939	0.930	0.048	[0.045;	0.051]	0.941	0.932
Metric invariance	0.049	[0.046;	0.051]	0.938	0.932	0.048	[0.045;	0.051]	0.941	0.935
Scalar invariance	0.049	[0.046;	0.052]	0.935	0.931	0.049	[0.046;	0.052]	0.935	0.932

Note. RMSEA = robust root mean square error of approximation; CFI = robust comparative fit index; TLI = robust Tucker-Lewis index; CI = confidence interval.

Table S3

Standardized and Unstandardized Coefficients for social-class$_{Education}$ (N = 2170) and social-class$_{Occupation}$ (N = 1802)

		Social-class$_{Education}$			Social-class$_{Occupation}$		
Observed variable	Latent construct	β	*B*	*SE*	β	*B*	*SE*
MAC_1	Independence	0.64	1.00		0.62	1.00	
MAC_3	Independence	0.79	1.02	0.05	0.82	1.16	0.05
MAC_4	Independence	0.51	0.98	0.06	0.50	1.01	0.07
MAC_5	Independence	0.60	0.97	0.05	0.57	0.98	0.05
MAC_8	Independence	0.57	0.86	0.04	0.55	0.87	0.05
MAC_10	Independence	0.76	1.01	0.05	0.78	1.07	0.05
MAC_13	Independence	0.45	0.84	0.05	0.65	0.87	0.06
MAC_2	Interdependence	0.71	1.00		0.71	1.00	
MAC_6	Interdependence	0.64	0.88	0.03	0.64	0.87	0.04
MAC_7	Interdependence	0.78	1.14	0.04	0.77	1.13	0.04
MAC_9	Interdependence	0.63	0.94	0.04	0.64	0.95	0.04
MAC_11	Interdependence	0.72	0.97	0.03	0.71	0.96	0.03
MAC_12	Interdependence	0.59	0.90	0.04	0.57	0.86	0.04
SOB_1	Sense of belonging	0.72	1.00		0.73	1.00	
SOB_2	Sense of belonging	0.83	1.09	0.04	0.81	1.06	0.05
SOB_3_rev	Sense of belonging	0.57	0.86	0.05	0.56	0.83	0.05
SE_1	Self-efficacy	0.78	1.00		0.78	1.00	
SE_2	Self-efficacy	0.83	0.98	0.02	0.83	1.01	0.02
SE_3	Self-efficacy	0.77	0.89	0.02	0.77	0.92	0.03
SE_4	Self-efficacy	0.79	0.83	0.02	0.79	0.83	0.02
SE_5	Self-efficacy	0.82	1.06	0.02	0.83	1.07	0.03
IDO_1	Intention to drop-out	0.89	1.00		0.86	1.00	
IDO_2	Intention to drop-out	0.85	0.99	0.02	0.85	0.98	0.02
IDO_3_rev	Intention to drop-out	0.30	0.35	0.03	0.30	0.34	0.03
IDO_4	Intention to drop-out	0.68	0.77	0.02	0.68	0.77	0.02

Note. β = standardized loadings, *B* = unstandardized loadings, *SE* = standard error. All items loaded significantly with $p < .001$ on the corresponding factors. Item IDO_3_rev was excluded from further analyses. See item coding at the project's Open Science Framework page (https://osf.io/qvk4n/?view_only=a264bfc041ff4c889dfff8772b38630e).

Table S4

Results of (robust) regression analyses for social-class$_{Education}$ (N = 2170) and social-class$_{Occupation}$ (N = 1802)

	Social-class$_{Education}$						Social-class$_{Occupation}$					
Variables	Estimate	[95% CI]	*SE*	*t*	*p*	η^2_p	Estimate	[95% CI]	*SE*	*t*	*p*	η^2_p
**1. Digital Equipment**												
Desktop computer: Number/Users												
Intercept	1.07	[0.98; 1.17]	0.05	23.16	<.001***		1.09	[0.99; 1.19]	0.05	22.43	<.001***	
Social-class	0.01	[-0.02; 0.03]	0.01	0.71	0.477	0.000	0.02	[-0.01; 0.05]	0.01	1.42	0.157	0.001
Year 2	-0.05	[-0.11; 0.00]	0.03	-1.89	0.059	0.002	-0.02	[-0.08; 0.05]	0.03	-0.55	0.581	0.001
Year 3	-0.01	[-0.07; 0.06]	0.03	-0.16	0.871	0.002	0.02	[-0.05; 0.09]	0.04	0.61	0.539	0.001
Gender	0.01	[-0.06; 0.09]	0.04	0.38	0.701	0.000	-0.02	[-0.10; 0.06]	0.04	-0.54	0.590	0.000
Intercept	1.08	[0.98; 1.17]	0.05	23.44	<.001***		1.09	[0.99; 1.20]	0.05	22.72	<.001***	0.000
Social-class	0.03	[-0.06; 0.13]	0.05	0.69	0.490	0.000	0.09	[-0.01; 0.20]	0.05	1.96	0.051	0.000
Year 2	-0.05	[-0.11; 0.01]	0.03	-1.75	0.080	0.002	-0.02	[-0.08; 0.05]	0.03	-0.54	0.590	0.001
Year 3	0.00	[-0.07; 0.06]	0.03	-0.08	0.935	0.002	0.02	[-0.05; 0.09]	0.04	0.6	0.547	0.001
Gender	0.01	[-0.07; 0.09]	0.04	0.25	0.803	0.000	-0.02	[-0.11; 0.06]	0.04	-0.64	0.523	0.000
Social-class × Year 2	0.02	[-0.04; 0.08]	0.03	0.71	0.475	0.000	-0.03	[-0.09; 0.04]	0.03	-0.78	0.435	0.001
Social-class × Year 3	0.01	[-0.05; 0.08]	0.03	0.37	0.712	0.000	-0.03	[-0.10; 0.04]	0.04	-0.88	0.378	0.001
Social-class × Gender	-0.03	[-0.11; 0.05]	0.04	-0.81	0.421	0.000	-0.05	[-0.14; 0.03]	0.04	-1.33	0.183	0.001
Intercept	1.07	[1.04; 1.09]	0.01	84.05	<.001***		1.07	[1.04; 1.09]	0.01	76.68	<.001***	
Social-class	0.01	[-0.01; 0.03]	0.01	0.78	0.433	0.000	0.02	[-0.01; 0.05]	0.01	1.42	0.155	0.001
Portable computer: Number/Users												
Intercept	1.81	[1.64; 1.99]	0.09	20.65	<.001***		1.77	[1.58; 1.96]	0.10	17.83	<.001***	
Social-class	0.10	[0.06; 0.15]	0.02	4.28	<.001***	0.009	0.11	[0.06; 0.16]	0.03	4.41	<.001***	0.011
Year 2	-0.11	[-0.22; -0.01]	0.05	-2.07	0.039	0.002	-0.16	[-0.28; -0.05]	0.06	-2.79	0.005**	0.005

	Social-class$_{Education}$						Social-class$_{Occupation}$					
Variables	Estimate	[95% CI]	*SE*	*t*	*p*	η^2_p	Estimate	[95% CI]	*SE*	*t*	*p*	η^2_p
Year 3	-0.08	[-0.20; 0.04]	0.06	-1.27	0.204	0.002	-0.03	[-0.16; 0.10]	0.07	-0.47	0.639	0.005
Gender	-0.10	[-0.25; 0.04]	0.07	-1.54	0.123	0.001	-0.08	[-0.23; 0.08]	0.07	-1.01	0.311	0.001
Intercept	1.79	[1.61; 1.97]	0.09	19.27	<.001***		1.75	[1.56; 1.94]	0.10	17.13	<.001***	
Social-class	-0.01	[-0.18; 0.17]	0.09	-0.07	0.941	0.000	-0.04	[-0.23; 0.15]	0.10	-0.41	0.680	0.000
Year 2	-0.11	[-0.22; 0.00]	0.06	-1.97	0.049	0.002	-0.17	[-0.29; -0.05]	0.06	-2.87	0.004**	0.005
Year 3	-0.09	[-0.21; 0.03]	0.06	-1.46	0.144	0.002	-0.04	[-0.17; 0.09]	0.07	-0.62	0.539	0.005
Gender	-0.08	[-0.22; 0.07]	0.07	-1.08	0.282	0.001	-0.05	[-0.21; 0.10]	0.08	-0.68	0.497	0.000
Social-class × Year 2	0.00	[-0.11; 0.11]	0.06	-0.02	0.985	0.001	-0.04	[-0.16; 0.07]	0.06	-0.75	0.451	0.001
Social-class × Year 3	-0.07	[-0.19; 0.05]	0.06	-1.09	0.276	0.001	-0.09	[-0.22; 0.04]	0.07	-1.34	0.182	0.001
Social-class × Gender	0.12	[-0.03; 0.26]	0.07	1.59	0.113	0.001	0.18	[0.02; 0.33]	0.08	2.21	0.027	0.003
Intercept	1.64	[1.59; 1.68]	0.02	67.20	<.001***		1.62	[1.57; 1.67]	0.03	62.57	<.001***	
Social-class	0.11	[0.06; 0.15]	0.02	4.39	<.001***	0.009	0.12	[0.07; 0.17]	0.03	4.47	<.001***	0.011
Tablet computer: Number/Users												
Intercept	1.22	[1.13; 1.32]	0.04	28.09	<.001***		1.21	[1.12; 1.31]	0.05	25.93	<.001***	
Social-class	-0.02	[-0.04; 0.01]	0.01	-1.33	0.185	0.001	0.01	[-0.02; 0.03]	0.01	0.57	0.566	0.000
Year 2	-0.07	[-0.12; -0.01]	0.03	-2.46	0.014	0.004	-0.04	[-0.10; 0.02]	0.03	-1.37	0.172	0.002
Year 3	-0.08	[-0.14; -0.01]	0.03	-2.34	0.019	0.004	-0.06	[-0.12; 0.01]	0.03	-1.61	0.108	0.002
Gender	-0.05	[-0.12; 0.03]	0.03	-1.43	0.154	0.001	-0.05	[-0.13; 0.03]	0.03	-1.58	0.114	0.001
Intercept	1.22	[1.13; 1.31]	0.04	27.75	<.001***		1.22	[1.12; 1.32]	0.05	25.99	<.001***	
Social-class	-0.04	[-0.14; 0.05]	0.04	-1.01	0.315	0.000	0.05	[-0.05; 0.14]	0.05	0.97	0.330	0.000
Year 2	-0.07	[-0.12; -0.01]	0.03	-2.30	0.021	0.003	-0.04	[-0.10; 0.02]	0.03	-1.36	0.173	0.002
Year 3	-0.07	[-0.13; -0.01]	0.03	-2.31	0.021	0.003	-0.05	[-0.12; 0.01]	0.03	-1.54	0.123	0.002
Gender	-0.05	[-0.12; 0.03]	0.03	-1.39	0.165	0.001	-0.06	[-0.14; 0.02]	0.03	-1.69	0.091	0.001

	Social-class$_{Education}$							Social-class$_{Occupation}$						
Variables	Estimate	[95% CI]	*SE*	*t*	*p*	η^2_p		Estimate	[95% CI]	*SE*	*t*	*p*	η^2_p	
Social-class × Year 2	0.03	[-0.03; 0.08]	0.03	0.94	0.347	0.000		-0.01	[-0.07; 0.05]	0.03	-0.43	0.666	0.000	
Social-class × Year 3	0.02	[-0.04; 0.08]	0.03	0.68	0.497	0.000		0.02	[-0.05; 0.08]	0.03	0.48	0.634	0.000	
Social-class × Gender	0.01	[-0.06; 0.09]	0.03	0.36	0.721	0.000		-0.03	[-0.11; 0.05]	0.03	-0.99	0.323	0.000	
Intercept	1.13	[1.10; 1.15]	0.01	92.49	<.001***			1.12	[1.10; 1.15]	0.01	85.09	<.001***		
Social-class	-0.01	[-0.04; 0.01]	0.01	-1.21	0.226	0.001		0.01	[-0.02; 0.03]	0.01	0.62	0.532	0.000	
Mobile Phone: Number/Users														
Intercept	2.51	[2.28; 2.74]	0.11	22.43	<.001***			2.48	[2.23; 2.74]	0.13	19.82	<.001***		
Social-class	0.03	[-0.03; 0.10]	0.03	1.09	0.277	0.001		0.03	[-0.04; 0.10]	0.03	0.82	0.410	0.000	
Year 2	-0.17	[-0.32; -0.03]	0.07	-2.41	0.016	0.004		-0.19	[-0.35; -0.04]	0.08	-2.42	0.016	0.004	
Year 3	-0.19	[-0.34; -0.03]	0.08	-2.36	0.018	0.004		-0.16	[-0.33; 0.01]	0.09	-1.84	0.066	0.004	
Gender	-0.28	[-0.47; -0.09]	0.09	-3.31	0.001**	0.004		-0.26	[-0.47; -0.05]	0.10	-2.74	0.006**	0.003	
Intercept	2.49	[2.26; 2.72]	0.11	21.67	<.001***			2.48	[2.22; 2.73]	0.13	19.50	<.001***		
Social-class	-0.03	[-0.26; 0.20]	0.11	-0.26	0.797	0.000		-0.08	[-0.34; 0.17]	0.13	-0.66	0.507	0.000	
Year 2	-0.17	[-0.31; -0.03]	0.07	-2.31	0.021	0.004		-0.19	[-0.35; -0.04]	0.08	-2.39	0.017	0.004	
Year 3	-0.20	[-0.35; -0.04]	0.08	-2.46	0.014	0.004		-0.17	[-0.34; 0.01]	0.09	-1.89	0.059	0.004	
Gender	-0.27	[-0.46; -0.08]	0.09	-3.02	0.003**	0.003		-0.25	[-0.46; -0.05]	0.10	-2.60	0.009**	0.003	
Social-class × Year 2	0.02	[-0.12; 0.16]	0.07	0.27	0.785	0.000		0.05	[-0.10; 0.21]	0.08	0.67	0.503	0.000	
Social-class × Year 3	-0.05	[-0.20; 0.11]	0.08	-0.57	0.570	0.000		-0.02	[-0.19; 0.15]	0.09	-0.28	0.783	0.000	
Social-class × Gender	0.06	[-0.13; 0.25]	0.09	0.69	0.493	0.000		0.09	[-0.12; 0.30]	0.10	0.91	0.363	0.000	
Intercept	2.08	[2.02; 2.14]	0.03	66.02	<.001***			2.08	[2.01; 2.15]	0.03	60.46	<.001***		
Social-class	0.04	[-0.02; 0.10]	0.03	1.21	0.225	0.001		0.03	[-0.04; 0.10]	0.03	0.91	0.365	0.000	
Access to IC[a]	Estimate	[95% *CI*]	*SE*	*z*	*p*	*OR*	[95% *CI*]	Estimate	[95% *CI*]	*SE*	*z*	*p*	*OR*	[95% *CI*]
Intercept	3.90	[0.64; 6.07]	0.00	6.07	<.001***			3.90	[2.64; 5.16]	0.64	6.07	<.001***		

	Social-class$_{Education}$							Social-class$_{Occupation}$						
Variables	Estimate	[95% CI]	*SE*	*t*	*p*	η^2_p		Estimate	[95% CI]	*SE*	*t*	*p*	η^2_p	
Social-class	0.02	[-0.31; 0.34]	0.17	0.10	0.920	1.02	[0.73; 1.41]	0.02	[-0.31; 0.34]	0.17	0.10	0.920	1.02	[0.73; 1.41]
Year 2	0.58	[-0.28; 1.45]	0.44	1.32	0.185	1.79	[0.76; 4.26]	0.58	[-0.28; 1.45]	0.44	1.32	0.185	1.79	[0.76; 4.26]
Year 3	-0.41	[-1.14; 0.31]	0.37	-1.11	0.265	0.66	[0.32; 1.37]	-0.41	[-1.14; 0.31]	0.37	-1.11	0.265	0.66	[0.32; 1.37]
Gender	0.09	[-0.96; 1.13]	0.53	0.16	0.870	1.09	[0.38; 3.11]	0.09	[-0.96; 1.13]	0.53	0.16	0.870	1.09	[0.38; 3.11]
Intercept	3.76	[2.38; 5.14]	0.70	5.34	<.001***			3.76	[2.38; 5.14]	0.70	5.34	<.001***		
Social-class	-0.53	[-1.92; 0.85]	0.70	-0.76	0.448	0.59	[0.15; 2.33]	-0.53	[-1.92; 0.85]	0.70	-0.76	0.448	0.59	[0.15; 2.33]
Year 2	0.60	[-0.29; 1.48]	0.45	1.32	0.186	1.81	[0.75; 4.39]	0.60	[-0.29; 1.48]	0.45	1.32	0.186	1.81	[0.75; 4.39]
Year 3	-0.42	[-1.15; 0.32]	0.37	-1.12	0.265	0.66	[0.32; 1.37]	-0.42	[-1.15; 0.32]	0.37	-1.12	0.265	0.66	[0.32; 1.37]
Gender	0.22	[-0.97; 1.42]	0.61	0.37	0.713	1.25	[0.38; 4.12]	0.22	[-0.97; 1.42]	0.61	0.37	0.713	1.25	[0.38; 4.12]
Social-class × Year 2	0.08	[-0.80; 0.97]	0.45	0.19	0.854	1.09	[0.45; 2.63]	0.08	[-0.80; 0.97]	0.45	0.19	0.854	1.09	[0.45; 2.63]
Social-class × Year 3	0.08	[-0.65; 0.81]	0.37	0.21	0.832	1.08	[0.52; 2.25]	0.08	[-0.65; 0.81]	0.37	0.21	0.832	1.08	[0.52; 2.25]
Social-class × Gender	0.46	[-0.73; 1.65]	0.61	0.76	0.450	1.58	[0.48; 5.21]	0.46	[-0.73; 1.65]	0.61	0.76	0.450	1.58	[0.48; 5.21]
Intercept	4.03	[3.70; 4.35]	0.17	24.31	<.001***			4.03	[3.70; 4.35]	0.17	24.31	<.001***		
Social-class	0.01	[-0.32; 0.33]	0.17	0.05	0.957	1.01	[0.73; 1.40]	0.01	[-0.32; 0.33]	0.17	0.05	0.957	1.01	[0.73; 1.40]
Access to HSI[a]	Estimate	[95% *CI*]	*SE*	*z*	*p*	*OR*	[95% *CI*]	Estimate	[95% *CI*]	*SE*	*z*	*p*	*OR*	[95% *CI*]
Intercept	0.27	[-0.06; 0.59]	0.17	1.60	0.109			0.27	[-0.06; 0.59]	0.17	1.60	0.109		
Social-class	0.04	[-0.05; 0.12]	0.04	0.81	0.420	1.04	[0.95; 1.13]	0.04	[-0.05; 0.12]	0.04	0.81	0.420	1.04	[0.95; 1.13]
Year 2	-0.30	[-0.50; -0.10]	0.10	-2.97	0.003**	0.74	[0.61; 0.90]	-0.30	[-0.50; -0.10]	0.10	-2.97	0.003**	0.74	[0.61; 0.90]
Year 3	-0.33	[-0.55; -0.12]	0.11	-3.01	0.003**	0.72	[0.58; 0.89]	-0.33	[-0.55; -0.12]	0.11	-3.01	0.003**	0.72	[0.58; 0.89]
Gender	0.16	[-0.10; 0.43]	0.14	1.20	0.230	1.18	[0.90; 1.54]	0.16	[-0.10; 0.43]	0.14	1.20	0.230	1.18	[0.90; 1.54]
Intercept	0.22	[-0.11; 0.55]	0.17	1.31	0.191			0.22	[-0.11; 0.55]	0.17	1.31	0.191		
Social-class	-0.19	[-0.52; 0.14]	0.17	-1.13	0.259	0.83	[0.59; 1.15]	-0.19	[-0.52; 0.14]	0.17	-1.13	0.259	0.83	[0.59; 1.15]
Year 2	-0.31	[-0.51; -0.11]	0.10	-2.99	0.003**	0.74	[0.60; 0.90]	-0.31	[-0.51; -0.11]	0.10	-2.99	0.003**	0.74	[0.60; 0.90]
Year 3	-0.35	[-0.57; -0.13]	0.11	-3.12	0.002**	0.70	[0.57; 0.88]	-0.35	[-0.57; -0.13]	0.11	-3.12	0.002**	0.70	[0.57; 0.88]

	Social-class$_{Education}$							Social-class$_{Occupation}$						
Variables	Estimate	[95% CI]	*SE*	*t*	*p*	η^2_p		Estimate	[95% CI]	*SE*	*t*	*p*	η^2_p	
Gender	0.21	[-0.07; 0.49]	0.14	1.50	0.135	1.23	[0.94; 1.62]	0.21	[-0.07; 0.49]	0.14	1.50	0.135	1.23	[0.94; 1.62]
Social-class × Year 2	-0.04	[-0.24; 0.17]	0.10	-0.34	0.733	0.97	[0.79; 1.18]	-0.04	[-0.24; 0.17]	0.10	-0.34	0.733	0.97	[0.79; 1.18]
Social-class × Year 3	-0.05	[-0.27; 0.17]	0.11	-0.44	0.661	0.95	[0.76; 1.19]	-0.05	[-0.27; 0.17]	0.11	-0.44	0.661	0.95	[0.76; 1.19]
Social-class × Gender	0.23	[-0.05; 0.50]	0.14	1.62	0.106	1.25	[0.95; 1.65]	0.23	[-0.05; 0.50]	0.14	1.62	0.106	1.25	[0.95; 1.65]
Intercept	0.25	[0.17; 0.34]	0.04	5.81	<.001***			0.25	[0.17; 0.34]	0.04	5.81	<.001***		
Social-class	0.04	[-0.05; 0.13]	0.04	0.92	0.358	1.04	[0.96; 1.13]	0.04	[-0.05; 0.13]	0.04	0.92	0.358	1.04	[0.96; 1.13]
**2. Material equipment**														
Desk to study[a]	Estimate	[95% *CI*]	*SE*	*z*	*p*	*OR*	[95% *CI*]	Estimate	[95% *CI*]	*SE*	*z*	*p*	*OR*	[95% *CI*]
Intercept	2.94	[2.28; 3.60]	0.34	8.73	<.001***			3.17	[2.45; 3.88]	0.36	8.71	<.001***		
Social-class	0.19	[0.01; 0.38]	0.09	2.06	0.039	1.21	[1.01; 1.46]	0.23	[0.03; 0.43]	0.10	2.30	0.022	1.265	[1.04; 1.54]
Year 2	-0.25	[-0.65; 0.16]	0.21	-1.20	0.231	0.78	[0.52; 1.17]	-0.26	[-0.71; 0.19]	0.23	-1.14	0.256	0.77	[0.49; 1.21]
Year 3	-0.10	[-0.56; 0.36]	0.23	-0.42	0.674	0.91	[0.57; 1.43]	-0.10	[-0.61; 0.40]	0.26	-0.41	0.686	0.90	[0.54; 1.49]
Gender	-0.06	[-0.59; 0.46]	0.27	-0.24	0.813	0.94	[0.55; 1.59]	-0.24	[-0.79; 0.31]	0.28	-0.85	0.397	0.79	[0.45; 1.37]
Intercept	3.00	[2.31; 3.70]	0.35	8.52	<.001***			3.24	[2.52; 3.96]	0.37	8.82	<.001***		
Social-class	0.39	[-0.30; 1.08]	0.35	1.10	0.269	1.48	[0.74; 2.95]	0.68	[-0.04; 1.40]	0.37	1.85	0.064	1.97	[0.96; 4.06]
Year 2	-0.33	[-0.76; 0.10]	0.22	-1.51	0.131	0.72	[0.47; 1.10]	-0.22	[-0.69; 0.25]	0.24	-0.92	0.356	0.80	[0.50; 1.28]
Year 3	-0.13	[-0.62; 0.36]	0.25	-0.51	0.614	0.88	[0.54; 1.44]	-0.10	[-0.62; 0.42]	0.27	-0.39	0.698	0.90	[0.54; 1.52]
Gender	-0.08	[-0.63; 0.47]	0.28	-0.29	0.770	0.92	[0.53; 1.60]	-0.31	[-0.87; 0.25]	0.28	-1.10	0.273	0.73	[0.42; 1.28]
Social-class × Year 2	-0.27	[-0.69; 0.16]	0.22	-1.22	0.223	0.77	[0.50; 1.18]	0.10	[-0.37; 0.57]	0.24	0.40	0.688	1.10	[0.69; 1.76]
Social-class × Year 3	-0.09	[-0.58; 0.40]	0.25	-0.38	0.707	0.91	[0.56; 1.49]	-0.05	[-0.57; 0.47]	0.27	-0.19	0.849	0.95	[0.57; 1.60]
Social-class × Gender	-0.06	[-0.61; 0.49]	0.28	-0.20	0.840	0.94	[0.54; 1.64]	-0.41	[-0.96; 0.15]	0.28	-1.44	0.150	0.66	[0.38; 1.16]
Intercept	2.75	[2.57; 2.93]	0.09	29.47	<.001***			2.77	[2.57; 2.97]	0.10	27.16	<.001***		
Social-class	0.20	[0.01; 0.38]	0.09	2.11	0.035	1.22	[1.01; 1.46]	0.24	[0.04; 0.44]	0.10	2.33	0.020	1.27	[1.04; 1.55]

	Social-class$_{Education}$							Social-class$_{Occupation}$						
Variables	Estimate	[95% CI]	*SE*	*t*	*p*	η^2_p		Estimate	[95% CI]	*SE*	*t*	*p*	η^2_p	
***Quiet place to study*[a]**	Estimate	[95% *CI*]	*SE*	*z*	*p*	*OR*	[95% *CI*]	Estimate	[95% *CI*]	*SE*	*z*	*p*	*OR*	[95% *CI*]
Intercept	1.68	[1.25; 2.11]	0.22	7.70	<.001***			1.59	[1.11; 2.07]	0.24	6.54	<.001***		
Social-class	0.16	[0.05; 0.28]	0.06	2.80	0.005**	1.18	[1.05; 1.32]	0.29	[0.17; 0.42]	0.06	4.61	<.001***	1.34	[1.18; 1.52]
Year 2	-0.25	[-0.51; 0.01]	0.13	-1.89	0.059	0.78	[0.60; 1.01]	-0.26	[-0.55; 0.02]	0.15	-1.81	0.070	0.77	[0.58; 1.02]
Year 3	-0.36	[-0.64; -0.08]	0.14	-2.53	0.011	0.70	[0.53; 0.92]	-0.27	[-0.58; 0.04]	0.16	-1.72	0.086	0.76	[0.56; 1.04]
Gender	0.06	[-0.29; 0.40]	0.18	0.32	0.750	1.06	[0.75; 1.50]	0.13	[-0.25; 0.52]	0.20	0.68	0.494	1.14	[0.78; 1.68]
Intercept	1.56	[1.09; 2.03]	0.24	6.46	<.001***			1.49	[0.97; 2.02]	0.27	5.56	<.001***		
Social-class	-0.22	[-0.69; 0.26]	0.24	-0.89	0.371	0.81	[0.50; 1.29]	0.09	[-0.44; 0.62]	0.27	0.33	0.741	1.09	[0.65; 1.85]
Year 2	-0.32	[-0.59; -0.05]	0.14	-2.34	0.020	0.73	[0.55; 0.95]	-0.36	[-0.66; -0.06]	0.15	-2.37	0.018	0.70	[0.52; 0.94]
Year 3	-0.35	[-0.65; -0.06]	0.15	-2.35	0.019	0.70	[0.52; 0.94]	-0.33	[-0.66; 0.00]	0.17	-1.96	0.050	0.72	[0.52; 1.00]
Gender	0.20	[-0.20; 0.59]	0.20	0.97	0.332	1.22	[0.82; 1.81]	0.28	[-0.16; 0.72]	0.23	1.25	0.212	1.33	[0.85; 2.06]
Social-class × Year 2	-0.28	[-0.54; -0.01]	0.14	-2.01	0.045	0.76	[0.58; 0.99]	-0.36	[-0.66; -0.06]	0.15	-2.35	0.019	0.70	[0.52; 0.94]
Social-class × Year 3	0.06	[-0.23; 0.36]	0.15	0.41	0.679	1.06	[0.79; 1.43]	-0.16	[-0.49; 0.17]	0.17	-0.95	0.345	0.85	[0.61; 1.19]
Social-class × Gender	0.42	[0.02; 0.82]	0.20	2.08	0.037	1.53	[1.03; 2.27]	0.36	[-0.09; 0.80]	0.23	1.58	0.114	1.43	[0.92; 2.22]
Intercept	1.55	[1.44; 1.67]	0.06	26.83	<.001***			1.57	[1.44; 1.69]	0.06	24.60	<.001***		
Social-class	0.17	[0.05; 0.28]	0.06	2.87	0.004**	1.18	[1.05; 1.32]	0.30	[0.17; 0.42]	0.06	4.63	<.001***	1.34	[1.19; 1.52]
**3. Digital use**														
f leisure activities														
Intercept	5.03	[4.78; 5.28]	0.13	39.68	<.001***			5.02	[4.74; 5.30]	0.14	36.45	<.001***		
Social-class	0.04	[-0.02; 0.11]	0.03	1.31	0.192	0.001		0.01	[-0.06; 0.09]	0.04	0.40	0.693	0.000	
Year 2	-0.05	[-0.21; 0.10]	0.08	-0.70	0.487	0.003		-0.03	[-0.20; 0.13]	0.08	-0.41	0.682	0.003	
Year 3	-0.21	[-0.38; -0.05]	0.09	-2.43	0.015	0.003		-0.20	[-0.38; -0.02]	0.10	-2.07	0.038	0.003	
Gender	0.27	[0.07; 0.48]	0.10	2.66	0.008**	0.003		0.27	[0.05; 0.50]	0.11	2.46	0.014	0.003	
Intercept	5.02	[4.77; 5.27]	0.13	39.85	<.001***			5.02	[4.74; 5.30]	0.14	36.03	<.001***		

	Social-class$_{Education}$						Social-class$_{Occupation}$					
Variables	Estimate	[95% CI]	*SE*	*t*	*p*	η^2_p	Estimate	[95% CI]	*SE*	*t*	*p*	η^2_p
Social-class	-0.06	[-0.31; 0.20]	0.13	-0.45	0.652	0.000	0.00	[-0.28; 0.27]	0.14	-0.01	0.988	0.000
Year 2	-0.05	[-0.20; 0.11]	0.08	-0.63	0.531	0.003	-0.03	[-0.20; 0.14]	0.08	-0.40	0.688	0.003
Year 3	-0.20	[-0.37; -0.03]	0.09	-2.31	0.021	0.003	-0.20	[-0.39; -0.02]	0.10	-2.08	0.038	0.003
Gender	0.28	[0.07; 0.49]	0.10	2.72	0.007**	0.003	0.27	[0.05; 0.50]	0.11	2.42	0.015	0.003
Social-class × Year 2	0.04	[-0.11; 0.20]	0.08	0.55	0.581	0.001	0.02	[-0.15; 0.18]	0.08	0.18	0.857	0.000
Social-class × Year 3	0.09	[-0.08; 0.26]	0.09	1.05	0.295	0.001	-0.01	[-0.20; 0.17]	0.10	-0.15	0.880	0.000
Social-class × Gender	0.06	[-0.15; 0.26]	0.10	0.55	0.586	0.000	0.01	[-0.21; 0.24]	0.11	0.12	0.906	0.000
Intercept	5.26	[5.19; 5.32]	0.03	154.99	<.001***		5.26	[5.19; 5.33]	0.04	142.19	<.001***	
Social-class	0.04	[-0.02; 0.11]	0.03	1.31	0.190	0.001	0.01	[-0.06; 0.09]	0.04	0.37	0.711	0.000
f university work												
Intercept	6.92	[6.76; 7.08]	0.10	67.98	<.001***		6.90	[6.72; 7.08]	0.12	58.87	<.001***	
Social-class	0.04	[0.00; 0.08]	0.02	1.88	0.060	0.002	0.04	[-0.01; 0.09]	0.02	1.62	0.105	0.001
Year 2	-0.01	[-0.11; 0.09]	0.05	-0.15	0.884	0.004	-0.01	[-0.12; 0.10]	0.06	-0.17	0.867	0.005
Year 3	0.14	[0.03; 0.24]	0.05	2.51	0.012	0.004	0.17	[0.05; 0.29]	0.06	2.78	0.005**	0.005
Gender	-0.53	[-0.66; -0.40]	0.09	-6.06	<.001***	0.028	-0.54	[-0.69; -0.39]	0.10	-5.31	<.001***	0.027
Intercept	6.92	[6.76; 7.09]	0.10	67.29	<.001***		6.90	[6.72; 7.08]	0.12	58.80	<.001***	
Social-class	0.04	[-0.13; 0.20]	0.10	0.36	0.719	0.000	-0.02	[-0.21; 0.16]	0.12	-0.20	0.845	0.000
Year 2	-0.02	[-0.12; 0.08]	0.05	-0.33	0.742	0.004	-0.01	[-0.12; 0.10]	0.06	-0.17	0.869	0.006
Year 3	0.14	[0.03; 0.25]	0.05	2.57	0.010	0.004	0.18	[0.05; 0.30]	0.06	2.84	0.005**	0.006
Gender	-0.53	[-0.67; -0.40]	0.09	-5.99	<.001***	0.027	-0.54	[-0.69; -0.39]	0.10	-5.30	<.001***	0.027
Social-class × Year 2	-0.06	[-0.16; 0.04]	0.05	-1.09	0.278	0.001	0.03	[-0.09; 0.14]	0.06	0.44	0.657	0.001
Social-class × Year 3	0.02	[-0.09; 0.12]	0.05	0.29	0.772	0.001	0.08	[-0.05; 0.20]	0.06	1.24	0.215	0.001
Social-class × Gender	0.02	[-0.12; 0.15]	0.09	0.20	0.842	0.000	0.03	[-0.12; 0.18]	0.10	0.30	0.768	0.000

	Social-class$_{Education}$						Social-class$_{Occupation}$					
Variables	Estimate	[95% CI]	*SE*	*t*	*p*	η^2_p	Estimate	[95% CI]	*SE*	*t*	*p*	η^2_p
Intercept	6.36	[6.31; 6.40]	0.02	289.06	<.001***		6.34	[6.29; 6.39]	0.02	254.64	<.001***	
Social-class	0.04	[0.00; 0.09]	0.02	1.93	0.054	0.002	0.04	[-0.01; 0.09]	0.02	1.71	0.088	0.002
f information search												
Intercept	5.20	[4.92; 5.48]	0.16	33.51	<.001***		5.21	[4.90; 5.52]	0.17	30.45	<.001***	
Social-class	0.01	[-0.07; 0.08]	0.04	0.25	0.799	0.000	0.01	[-0.07; 0.09]	0.04	0.21	0.832	0.000
Year 2	0.09	[-0.09; 0.26]	0.09	0.95	0.342	0.027	0.14	[-0.05; 0.33]	0.10	1.41	0.157	0.027
Year 3	0.71	[0.52; 0.90]	0.09	7.51	<.001***	0.027	0.73	[0.52; 0.93]	0.10	7.02	<.001***	0.027
Gender	-0.44	[-0.68; -0.21]	0.13	-3.38	0.001**	0.007	-0.45	[-0.70; -0.20]	0.15	-3.07	0.002**	0.007
Intercept	5.20	[4.92; 5.49]	0.16	32.62	<.001***		5.22	[4.91; 5.53]	0.17	30.24	<.001***	
Social-class	0.01	[-0.27; 0.30]	0.16	0.09	0.928	0.000	0.06	[-0.25; 0.37]	0.17	0.34	0.732	0.000
Year 2	0.11	[-0.07; 0.28]	0.09	1.16	0.245	0.026	0.14	[-0.05; 0.33]	0.10	1.45	0.147	0.027
Year 3	0.71	[0.52; 0.90]	0.10	7.37	<.001***	0.026	0.73	[0.52; 0.94]	0.10	7.02	<.001***	0.027
Gender	-0.45	[-0.68; -0.21]	0.14	-3.30	0.001**	0.006	-0.46	[-0.71; -0.20]	0.15	-3.10	0.002**	0.007
Social-class × Year 2	0.12	[-0.05; 0.30]	0.09	1.36	0.173	0.001	0.05	[-0.14; 0.23]	0.10	0.46	0.645	0.000
Social-class × Year 3	0.01	[-0.18; 0.20]	0.10	0.12	0.905	0.001	0.04	[-0.17; 0.25]	0.10	0.38	0.705	0.000
Social-class × Gender	-0.05	[-0.28; 0.19]	0.14	-0.34	0.735	0.000	-0.07	[-0.32; 0.18]	0.15	-0.47	0.642	0.000
Intercept	4.92	[4.84; 4.99]	0.04	125.90	<.001***		4.95	[4.87; 5.03]	0.04	117.09	<.001***	
Social-class	0.01	[-0.07; 0.09]	0.04	0.23	0.815	0.000	0.01	[-0.07; 0.09]	0.04	0.21	0.836	0.000
f staying in contact												
Intercept	5.94	[5.68; 6.19]	0.14	42.54	<.001***		5.83	[5.56; 6.10]	0.15	39.80	<.001***	
Social-class	0.04	[-0.02; 0.11]	0.03	1.30	0.194	0.001	0.08	[0.00; 0.15]	0.04	2.06	0.040	0.002
Year 2	0.04	[-0.12; 0.19]	0.08	0.47	0.639	0.001	0.10	[-0.07; 0.27]	0.09	1.17	0.241	0.001
Year 3	0.10	[-0.07; 0.28]	0.09	1.19	0.234	0.001	0.11	[-0.07; 0.29]	0.09	1.20	0.232	0.001

	Social-class$_{Education}$						Social-class$_{Occupation}$					
Variables	Estimate	[95% CI]	*SE*	*t*	*p*	η^2_p	Estimate	[95% CI]	*SE*	*t*	*p*	η^2_p
Gender	-0.47	[-0.68; -0.26]	0.12	-4.03	<.001***	0.009	-0.37	[-0.59; -0.15]	0.12	-3.06	0.002**	0.006
Intercept	5.92	[5.66; 6.18]	0.14	42.82	<.001***		5.83	[5.56; 6.11]	0.15	39.92	<.001***	
Social-class	-0.18	[-0.44; 0.08]	0.14	-1.33	0.185	0.000	0.02	[-0.26; 0.29]	0.15	0.13	0.895	0.000
Year 2	0.05	[-0.11; 0.20]	0.08	0.55	0.580	0.001	0.11	[-0.06; 0.27]	0.09	1.23	0.219	0.001
Year 3	0.12	[-0.05; 0.30]	0.09	1.40	0.162	0.001	0.12	[-0.07; 0.30]	0.09	1.22	0.221	0.001
Gender	-0.46	[-0.67; -0.24]	0.12	-3.95	<.001***	0.008	-0.38	[-0.60; -0.15]	0.12	-3.11	0.002**	0.006
Social-class × Year 2	0.07	[-0.08; 0.23]	0.08	0.91	0.365	0.002	0.09	[-0.08; 0.26]	0.09	1.08	0.280	0.001
Social-class × Year 3	0.18	[0.01; 0.36]	0.09	2.09	0.036	0.002	0.07	[-0.12; 0.25]	0.09	0.72	0.474	0.001
Social-class × Gender	0.14	[-0.07; 0.35]	0.12	1.21	0.228	0.001	0.00	[-0.22; 0.23]	0.12	0.04	0.970	0.000
Intercept	5.45	[5.38; 5.52]	0.03	157.11	<.001***		5.48	[5.41; 5.55]	0.04	149.62	<.001***	
Social-class	0.05	[-0.02; 0.11]	0.03	1.31	0.190	0.001	0.08	[0.00; 0.15]	0.04	2.09	0.037	0.002
**4. Self-construal**												
Independence												
Intercept	5.78	[5.65; 5.92]	0.07	84.73	<.001***		5.78	[5.64; 5.93]	0.08	75.82	<.001***	
Social-class	0.06	[0.02; 0.09]	0.02	3.17	0.002**	0.005	0.06	[0.02; 0.10]	0.02	3.10	0.002**	0.005
Year 2	-0.05	[-0.13; 0.04]	0.04	-1.12	0.264	0.001	0.00	[-0.09; 0.09]	0.04	0.04	0.966	0.000
Year 3	-0.05	[-0.14; 0.04]	0.05	-1.07	0.283	0.001	-0.03	[-0.12; 0.07]	0.05	-0.58	0.561	0.000
Gender	-0.08	[-0.18; 0.03]	0.06	-1.34	0.180	0.001	-0.09	[-0.21; 0.03]	0.06	-1.44	0.151	0.001
Intercept	5.79	[5.65; 5.92]	0.07	84.41	<.001***		5.78	[5.63; 5.92]	0.08	75.72	<.001***	
Social-class	0.04	[-0.10; 0.17]	0.07	0.54	0.587	0.000	-0.07	[-0.21; 0.08]	0.08	-0.89	0.373	0.000
Year 2	-0.04	[-0.13; 0.04]	0.04	-1.10	0.273	0.001	0.00	[-0.09; 0.09]	0.04	0.01	0.994	0.000
Year 3	-0.04	[-0.13; 0.05]	0.05	-0.91	0.361	0.001	-0.03	[-0.12; 0.07]	0.05	-0.56	0.574	0.000
Gender	-0.08	[-0.19; 0.03]	0.06	-1.39	0.166	0.001	-0.08	[-0.20; 0.03]	0.06	-1.33	0.182	0.001

	Social-class$_{Education}$						Social-class$_{Occupation}$					
Variables	Estimate	[95% CI]	*SE*	*t*	*p*	η^2_p	Estimate	[95% CI]	*SE*	*t*	*p*	η^2_p
Social-class × Year 2	0.01	[-0.07; 0.09]	0.04	0.30	0.763	0.001	0.03	[-0.06; 0.11]	0.04	0.57	0.568	0.001
Social-class × Year 3	0.05	[-0.04; 0.14]	0.05	1.17	0.243	0.001	0.06	[-0.03; 0.16]	0.05	1.31	0.191	0.001
Social-class × Gender	0.00	[-0.11; 0.11]	0.06	0.01	0.993	0.000	0.09	[-0.03; 0.21]	0.06	1.43	0.153	0.001
Intercept	5.67	[5.64; 5.71]	0.02	321.75	<.001***		5.68	[5.64; 5.71]	0.02	297.11	<.001***	
Social-class	0.06	[0.02; 0.09]	0.02	3.23	0.001**	0.005	0.06	[0.02; 0.10]	0.02	3.13	0.002**	0.005
Interdependence												
Intercept	4.56	[4.35; 4.78]	0.11	40.38	<.001***		4.61	[4.38; 4.85]	0.12	37.19	<.001***	
Social-class	-0.23	[-0.29; -0.18]	0.03	-7.94	<.001***	0.029	-0.24	[-0.30; -0.18]	0.03	-7.63	<.001***	0.032
Year 2	-0.10	[-0.23; 0.03]	0.07	-1.49	0.138	0.002	-0.05	[-0.19; 0.09]	0.07	-0.66	0.508	0.001
Year 3	-0.12	[-0.27; 0.02]	0.07	-1.64	0.102	0.002	-0.09	[-0.25; 0.06]	0.08	-1.18	0.236	0.001
Gender	-0.11	[-0.29; 0.06]	0.09	-1.19	0.233	0.001	-0.10	[-0.29; 0.09]	0.10	-1.01	0.315	0.001
Intercept	4.56	[4.34; 4.78]	0.11	39.84	<.001***		4.62	[4.39; 4.85]	0.12	37.14	<.001***	
Social-class	-0.29	[-0.51; -0.07]	0.11	-2.52	0.012	0.000	-0.32	[-0.55; -0.08]	0.12	-2.53	0.011	0.000
Year 2	-0.10	[-0.23; 0.04]	0.07	-1.40	0.162	0.001	-0.04	[-0.18; 0.10]	0.07	-0.56	0.578	0.001
Year 3	-0.11	[-0.26; 0.04]	0.07	-1.49	0.137	0.001	-0.09	[-0.24; 0.07]	0.08	-1.08	0.280	0.001
Gender	-0.11	[-0.29; 0.07]	0.10	-1.20	0.231	0.001	-0.12	[-0.31; 0.08]	0.10	-1.11	0.268	0.001
Social-class × Year 2	0.04	[-0.10; 0.17]	0.07	0.54	0.588	0.001	0.14	[0.00; 0.29]	0.07	1.94	0.052	0.003
Social-class × Year 3	0.09	[-0.06; 0.23]	0.07	1.17	0.240	0.001	0.16	[0.00; 0.31]	0.08	1.96	0.050	0.003
Social-class × Gender	0.02	[-0.16; 0.20]	0.10	0.18	0.860	0.000	-0.01	[-0.21; 0.18]	0.10	-0.14	0.886	0.000
Intercept	4.37	[4.31; 4.43]	0.03	148.81	<.001***		4.45	[4.39; 4.52]	0.03	141.53	<.001***	
Social-class	-0.23	[-0.29; -0.17]	0.03	-7.90	<.001***	0.028	-0.24	[-0.30; -0.18]	0.03	-7.60	<.001***	0.031
**5. Psychological factors**												
Sense of belonging												

	Social-class$_{Education}$						Social-class$_{Occupation}$					
Variables	Estimate	[95% CI]	*SE*	*t*	*p*	η^2_p	Estimate	[95% CI]	*SE*	*t*	*p*	η^2_p
Intercept	4.75	[4.54; 4.95]	0.10	45.49	<.001***		4.75	[4.53; 4.97]	0.11	42.78	<.001***	
Social-class	0.03	[-0.02; 0.09]	0.03	1.22	0.222	0.001	0.04	[-0.02; 0.09]	0.03	1.18	0.237	0.001
Year 2	-0.11	[-0.24; 0.01]	0.06	-1.81	0.070	0.002	-0.12	[-0.25; 0.02]	0.07	-1.77	0.077	0.003
Year 3	-0.12	[-0.26; 0.02]	0.07	-1.68	0.094	0.002	-0.14	[-0.29; 0.01]	0.08	-1.81	0.070	0.003
Gender	-0.07	[-0.24; 0.09]	0.08	-0.87	0.383	0.000	-0.05	[-0.23; 0.12]	0.09	-0.62	0.538	0.000
Intercept	4.75	[4.54; 4.96]	0.11	45.23	<.001***		4.76	[4.54; 4.98]	0.11	42.58	<.001***	
Social-class	0.01	[-0.19; 0.22]	0.11	0.14	0.892	0.000	0.10	[-0.12; 0.32]	0.11	0.91	0.364	0.000
Year 2	-0.12	[-0.25; 0.01]	0.06	-1.90	0.058	0.002	-0.12	[-0.25; 0.02]	0.07	-1.76	0.078	0.002
Year 3	-0.11	[-0.25; 0.03]	0.07	-1.56	0.119	0.002	-0.13	[-0.28; 0.01]	0.08	-1.71	0.087	0.002
Gender	-0.08	[-0.25; 0.09]	0.09	-0.90	0.369	0.000	-0.06	[-0.24; 0.12]	0.09	-0.70	0.482	0.000
Social-class × Year 2	-0.02	[-0.15; 0.10]	0.06	-0.38	0.706	0.001	-0.02	[-0.16; 0.11]	0.07	-0.36	0.720	0.001
Social-class × Year 3	0.06	[-0.08; 0.20]	0.07	0.85	0.398	0.001	0.08	[-0.07; 0.22]	0.08	0.99	0.322	0.001
Social-class × Gender	0.01	[-0.16; 0.18]	0.09	0.13	0.897	0.000	-0.07	[-0.25; 0.11]	0.09	-0.77	0.439	0.000
Intercept	4.59	[4.54; 4.65]	0.03	167.24	<.001***		4.61	[4.55; 4.67]	0.03	155.79	<.001***	
Social-class	0.04	[-0.02; 0.09]	0.03	1.31	0.192	0.001	0.04	[-0.02; 0.09]	0.03	1.23	0.221	0.001
Self-efficacy												
Intercept	4.77	[4.55; 4.99]	0.11	42.32	<.001***		4.75	[4.52; 4.99]	0.13	37.77	<.001***	
Social-class	0.02	[-0.04; 0.07]	0.03	0.59	0.554	0.000	0.05	[-0.01; 0.11]	0.03	1.54	0.124	0.001
Year 2	-0.27	[-0.40; -0.13]	0.07	-3.97	<.001***	0.008	-0.18	[-0.32; -0.03]	0.07	-2.39	0.017	0.004
Year 3	-0.21	[-0.35; -0.06]	0.07	-2.75	0.006**	0.008	-0.18	[-0.33; -0.02]	0.08	-2.13	0.033	0.004
Gender	-0.03	[-0.21; 0.14]	0.09	-0.35	0.725	0.000	-0.04	[-0.24; 0.15]	0.10	-0.42	0.673	0.000
Intercept	4.77	[4.56; 4.99]	0.11	41.64	<.001***		4.75	[4.51; 4.99]	0.13	37.23	<.001***	
Social-class	0.02	[-0.20; 0.24]	0.11	0.20	0.839	0.000	0.06	[-0.18; 0.30]	0.13	0.50	0.616	0.000
Year 2	-0.27	[-0.41; -0.14]	0.07	-4.01	<.001***	0.008	-0.18	[-0.33; -0.03]	0.07	-2.43	0.015	0.004

	Social-class$_{Education}$						Social-class$_{Occupation}$					
Variables	Estimate	[95% CI]	*SE*	*t*	*p*	η^2_p	Estimate	[95% CI]	*SE*	*t*	*p*	η^2_p
Year 3	-0.20	[-0.35; -0.05]	0.08	-2.63	0.009**	0.008	-0.18	[-0.34; -0.02]	0.08	-2.17	0.030	0.004
Gender	-0.04	[-0.22; 0.14]	0.10	-0.39	0.696	0.000	-0.04	[-0.23; 0.16]	0.11	-0.36	0.721	0.000
Social-class × Year 2	-0.03	[-0.16; 0.10]	0.07	-0.45	0.653	0.000	-0.06	[-0.20; 0.09]	0.07	-0.77	0.440	0.000
Social-class × Year 3	0.04	[-0.11; 0.19]	0.08	0.54	0.591	0.000	-0.07	[-0.22; 0.09]	0.08	-0.79	0.430	0.000
Social-class × Gender	0.00	[-0.18; 0.17]	0.10	-0.05	0.960	0.000	0.02	[-0.17; 0.22]	0.11	0.20	0.844	0.000
Intercept	4.58	[4.53; 4.64]	0.03	155.98	<.001***		4.60	[4.53; 4.66]	0.03	143.14	<.001***	
Social-class	0.02	[-0.04; 0.08]	0.03	0.76	0.448	0.000	0.05	[-0.01; 0.11]	0.03	1.59	0.113	0.001
Intention to drop-out												
Intercept	3.48	[3.20; 3.77]	0.14	24.32	<.001***		3.46	[3.14; 3.77]	0.16	22.09	<.001***	
Social-class	-0.16	[-0.24; -0.09]	0.04	-4.23	<.001***	0.008	-0.25	[-0.33; -0.17]	0.04	-6.03	<.001***	0.020
Year 2	0.17	[-0.01; 0.34]	0.09	1.89	0.059	0.006	0.21	[0.02; 0.40]	0.10	2.19	0.029	0.005
Year 3	0.34	[0.15; 0.53]	0.10	3.50	<.001***	0.006	0.32	[0.11; 0.52]	0.11	2.94	0.003**	0.005
Gender	0.03	[-0.21; 0.26]	0.11	0.24	0.810	0.000	0.06	[-0.20; 0.31]	0.12	0.45	0.653	0.000
Intercept	3.48	[3.19; 3.76]	0.14	24.25	<.001***		3.45	[3.13; 3.76]	0.16	21.84	<.001***	
Social-class	-0.12	[-0.41; 0.17]	0.14	-0.82	0.413	0.000	-0.27	[-0.59; 0.04]	0.16	-1.73	0.084	0.000
Year 2	0.16	[-0.02; 0.33]	0.09	1.77	0.078	0.005	0.21	[0.02; 0.40]	0.10	2.18	0.030	0.005
Year 3	0.32	[0.12; 0.51]	0.10	3.22	0.001**	0.005	0.31	[0.10; 0.52]	0.11	2.84	0.005	0.005
Gender	0.04	[-0.19; 0.28]	0.11	0.38	0.707	0.000	0.07	[-0.19; 0.32]	0.13	0.52	0.604	0.000
Social-class × Year 2	-0.09	[-0.26; 0.09]	0.09	-1.00	0.320	0.002	-0.01	[-0.20; 0.18]	0.10	-0.06	0.950	0.001
Social-class × Year 3	-0.18	[-0.38; 0.01]	0.10	-1.86	0.063	0.002	-0.14	[-0.35; 0.07]	0.11	-1.30	0.194	0.001
Social-class × Gender	0.03	[-0.21; 0.27]	0.11	0.26	0.793	0.000	0.05	[-0.20; 0.31]	0.13	0.42	0.673	0.000
Intercept	3.66	[3.59; 3.74]	0.04	95.68	<.001***		3.68	[3.59; 3.76]	0.04	87.89	<.001***	
Social-class	0.00	[-0.24; -0.09]	0.04	-4.31	<.001***	0.008	0.00	[-0.34; -0.17]	0.04	-6.08	<.001***	0.020

	Social-class$_{Education}$						Social-class$_{Occupation}$					
Variables	Estimate	[95% CI]	*SE*	*t*	*p*	η^2_p	Estimate	[95% CI]	*SE*	*t*	*p*	η^2_p
**6. Class attendance**												
Attending online class												
Intercept	6.14	[5.87; 6.40]	0.15	41.79	<.001***		6.16	[5.86; 6.46]	0.17	36.31	<.001***	
Social-class	0.02	[-0.05; 0.09]	0.04	0.49	0.622	0.000	0.05	[-0.03; 0.13]	0.04	1.16	0.247	0.001
Year 2	0.17	[0.01; 0.33]	0.08	2.03	0.043	0.007	0.18	[-0.01; 0.36]	0.10	1.81	0.071	0.010
Year 3	0.36	[0.18; 0.54]	0.09	4.05	<.001***	0.007	0.44	[0.24; 0.64]	0.10	4.47	<.001***	0.010
Gender	-0.34	[-0.56; -0.13]	0.12	-2.83	0.005**	0.004	-0.42	[-0.67; -0.18]	0.14	-3.02	0.003**	0.006
Intercept	6.13	[5.86; 6.40]	0.15	41.17	<.001***		6.16	[5.86; 6.47]	0.17	36.09	<.001***	
Social-class	-0.07	[-0.34; 0.20]	0.15	-0.49	0.625	0.000	0.07	[-0.23; 0.37]	0.17	0.42	0.674	0.000
Year 2	0.17	[0.01; 0.33]	0.09	2.00	0.045	0.008	0.18	[-0.01; 0.36]	0.10	1.81	0.070	0.011
Year 3	0.37	[0.19; 0.55]	0.09	4.12	<.001***	0.008	0.45	[0.25; 0.65]	0.10	4.55	<.001***	0.011
Gender	-0.34	[-0.56; -0.12]	0.12	-2.74	0.006**	0.004	-0.43	[-0.68; -0.18]	0.14	-3.04	0.002**	0.006
Social-class × Year 2	0.01	[-0.15; 0.17]	0.09	0.10	0.917	0.001	0.00	[-0.18; 0.18]	0.10	0.00	1.000	0.001
Social-class × Year 3	0.10	[-0.08; 0.28]	0.09	1.07	0.287	0.001	0.13	[-0.08; 0.33]	0.10	1.26	0.206	0.001
Social-class × Gender	0.06	[-0.16; 0.28]	0.12	0.45	0.649	0.000	-0.05	[-0.30; 0.20]	0.14	-0.36	0.715	0.000
Intercept	5.91	[5.84; 5.98]	0.04	164.10	<.001***		5.86	[5.78; 5.94]	0.04	144.03	<.001***	
Social-class	0.02	[-0.06; 0.09]	0.04	0.43	0.669	0.000	0.05	[-0.03; 0.13]	0.04	1.16	0.246	0.001
Being late for online class												
Intercept	5.88	[5.61; 6.15]	0.14	41.70	<.001***		5.94	[5.64; 6.23]	0.16	38.03	<.001***	
Social-class	-0.02	[-0.09; 0.05]	0.04	-0.62	0.534	0.000	0.02	[-0.06; 0.10]	0.04	0.56	0.576	0.000
Year 2	-0.09	[-0.25; 0.08]	0.08	-1.03	0.301	0.002	-0.02	[-0.20; 0.16]	0.09	-0.23	0.822	0.001
Year 3	-0.16	[-0.34; 0.02]	0.09	-1.76	0.079	0.002	-0.11	[-0.31; 0.08]	0.10	-1.11	0.266	0.001
Gender	-0.17	[-0.39; 0.05]	0.11	-1.53	0.126	0.001	-0.26	[-0.50; -0.02]	0.13	-2.07	0.039	0.003
Intercept	5.86	[5.59; 6.13]	0.14	41.00	<.001***		5.93	[5.64; 6.23]	0.16	37.72	<.001***	

	Social-class$_{Education}$						Social-class$_{Occupation}$					
Variables	Estimate	[95% CI]	*SE*	*t*	*p*	η^2_p	Estimate	[95% CI]	*SE*	*t*	*p*	η^2_p
Social-class	0.01	[-0.26; 0.28]	0.14	0.06	0.950	0.000	0.19	[-0.10; 0.49]	0.16	1.23	0.220	0.000
Year 2	-0.10	[-0.27; 0.06]	0.08	-1.21	0.228	0.002	-0.02	[-0.20; 0.16]	0.09	-0.26	0.794	0.001
Year 3	-0.20	[-0.38; -0.02]	0.09	-2.08	0.038	0.002	-0.13	[-0.33; 0.07]	0.10	-1.27	0.204	0.001
Gender	-0.15	[-0.37; 0.07]	0.12	-1.29	0.198	0.001	-0.25	[-0.49; -0.01]	0.13	-1.97	0.049	0.002
Social-class × Year 2	-0.12	[-0.29; 0.04]	0.08	-1.47	0.141	0.003	-0.12	[-0.30; 0.06]	0.09	-1.29	0.198	0.005
Social-class × Year 3	-0.23	[-0.42; -0.05]	0.09	-2.46	0.014	0.003	-0.31	[-0.51; -0.12]	0.10	-3.06	0.002**	0.005
Social-class × Gender	0.06	[-0.16; 0.29]	0.12	0.55	0.580	0.000	-0.04	[-0.28; 0.20]	0.13	-0.33	0.740	0.000
Intercept	5.61	[5.54; 5.68]	0.04	154.17	<.001***		5.61	[5.53; 5.69]	0.04	142.49	<.001***	
Social-class	-0.02	[-0.09; 0.05]	0.04	-0.57	0.572	0.000	0.02	[-0.05; 0.10]	0.04	0.61	0.541	0.000
Missing online class												
Intercept	5.78	[5.51; 6.06]	0.15	39.32	<.001***		5.83	[5.52; 6.13]	0.16	35.62	<.001***	
Social-class	-0.01	[-0.08; 0.07]	0.04	-0.17	0.864	0.000	0.02	[-0.06; 0.10]	0.04	0.54	0.589	0.000
Year 2	-0.42	[-0.59; -0.26]	0.09	-5.00	<.001***	0.014	-0.35	[-0.53; -0.16]	0.09	-3.70	<.001***	0.010
Year 3	-0.40	[-0.59; -0.22]	0.10	-4.22	<.001***	0.014	-0.36	[-0.56; -0.16]	0.10	-3.43	<.001***	0.010
Gender	-0.18	[-0.41; 0.04]	0.12	-1.52	0.130	0.001	-0.25	[-0.50; 0.00]	0.13	-1.88	0.061	0.002
Intercept	5.79	[5.51; 6.07]	0.15	39.14	<.001***		5.83	[5.52; 6.13]	0.16	35.39	<.001***	
Social-class	0.06	[-0.22; 0.34]	0.15	0.38	0.704	0.000	0.09	[-0.22; 0.39]	0.16	0.54	0.586	0.000
Year 2	-0.43	[-0.60; -0.25]	0.09	-4.98	<.001***	0.013	-0.35	[-0.53; -0.16]	0.09	-3.70	<.001***	0.010
Year 3	-0.41	[-0.60; -0.22]	0.10	-4.28	<.001***	0.013	-0.36	[-0.56; -0.16]	0.10	-3.45	0.001**	0.010
Gender	-0.18	[-0.41; 0.05]	0.12	-1.51	0.131	0.001	-0.25	[-0.50; 0.00]	0.13	-1.86	0.063	0.002
Social-class × Year 2	-0.01	[-0.18; 0.16]	0.09	-0.14	0.888	0.000	-0.03	[-0.22; 0.15]	0.09	-0.34	0.735	0.000
Social-class × Year 3	-0.08	[-0.26; 0.11]	0.10	-0.78	0.436	0.000	-0.07	[-0.27; 0.13]	0.10	-0.66	0.509	0.000
Social-class × Gender	-0.03	[-0.27; 0.20]	0.12	-0.29	0.773	0.000	-0.03	[-0.28; 0.21]	0.13	-0.26	0.798	0.000

	Social-class$_{Education}$						Social-class$_{Occupation}$					
Variables	Estimate	[95% CI]	*SE*	*t*	*p*	η^2_p	Estimate	[95% CI]	*SE*	*t*	*p*	η^2_p
Intercept	5.32	[5.25; 5.40]	0.04	141.70	<.001***		5.33	[5.25; 5.41]	0.04	130.47	<.001***	
Social-class	0.00	[-0.07; 0.08]	0.04	0.05	0.960	0.000	0.03	[-0.05; 0.11]	0.04	0.64	0.525	0.000
Attending on-campus class												
Intercept	2.46	[2.15; 2.77]	0.16	15.28	<.001***		2.35	[2.01; 2.69]	0.18	13.01	<.001***	
Social-class	0.05	[-0.04; 0.13]	0.04	1.09	0.277	0.001	0.02	[-0.07; 0.11]	0.05	0.45	0.653	0.000
Year 2	-0.20	[-0.39; -0.01]	0.10	-2.03	0.042	0.016	-0.21	[-0.41; 0.00]	0.11	-1.88	0.061	0.015
Year 3	-0.63	[-0.84; -0.42]	0.10	-6.16	<.001***	0.016	-0.61	[-0.84; -0.38]	0.11	-5.43	<.001***	0.015
Gender	0.23	[-0.02; 0.49]	0.13	1.80	0.073	0.002	0.31	[0.03; 0.59]	0.15	2.13	0.034	0.003
Intercept	2.43	[2.11; 2.74]	0.16	14.83	<.001***		2.33	[1.99; 2.68]	0.18	12.82	<.001***	
Social-class	-0.05	[-0.37; 0.26]	0.16	-0.33	0.745	0.000	-0.18	[-0.53; 0.16]	0.18	-1.01	0.315	0.000
Year 2	-0.21	[-0.40; -0.02]	0.10	-2.10	0.036	0.017	-0.21	[-0.42; 0.00]	0.11	-1.87	0.061	0.016
Year 3	-0.66	[-0.87; -0.45]	0.10	-6.37	<.001***	0.017	-0.63	[-0.85; -0.40]	0.11	-5.55	<.001***	0.016
Gender	0.27	[0.01; 0.53]	0.13	2.03	0.043	0.002	0.33	[0.05; 0.61]	0.15	2.23	0.026	0.003
Social-class × Year 2	-0.08	[-0.27; 0.11]	0.10	-0.77	0.440	0.001	0.08	[-0.13; 0.29]	0.11	0.70	0.486	0.001
Social-class × Year 3	-0.18	[-0.39; 0.03]	0.10	-1.74	0.082	0.001	-0.11	[-0.34; 0.12]	0.11	-0.99	0.320	0.001
Social-class × Gender	0.15	[-0.10; 0.41]	0.13	1.16	0.247	0.001	0.18	[-0.10; 0.46]	0.15	1.23	0.217	0.001
Intercept	2.49	[2.40; 2.57]	0.04	58.39	<.001***		2.47	[2.38; 2.56]	0.05	53.29	<.001***	
Social-class	0.05	[-0.03; 0.13]	0.04	1.15	0.249	0.001	0.02	[-0.07; 0.11]	0.05	0.46	0.643	0.000
Being late for on-campus class												
Intercept	1.17	[0.95; 1.38]	0.13	9.26	<.001***		1.18	[0.94; 1.43]	0.14	8.59	<.001***	
Social-class	0.06	[0.01; 0.12]	0.03	2.17	0.030	0.002	0.06	[-0.01; 0.12]	0.03	1.75	0.081	0.002
Year 2	0.19	[0.06; 0.32]	0.06	2.98	0.003**	0.008	0.23	[0.08; 0.38]	0.07	3.20	0.001**	0.010
Year 3	0.29	[0.15; 0.44]	0.08	3.81	<.001***	0.008	0.33	[0.16; 0.49]	0.09	3.78	<.001***	0.010

	Social-class$_{Education}$						Social-class$_{Occupation}$					
Variables	Estimate	[95% CI]	*SE*	*t*	*p*	η^2_p	Estimate	[95% CI]	*SE*	*t*	*p*	η^2_p
Gender	0.33	[0.15; 0.51]	0.11	3.09	0.002**	0.006	0.31	[0.12; 0.51]	0.12	2.73	0.006**	0.005
Intercept	6.84	[6.63; 7.06]	0.13	53.08	<.001***		6.83	[6.58; 7.07]	0.14	48.89	<.001***	
Social-class	0.03	[-0.19; 0.25]	0.13	0.23	0.814	0.000	0.07	[-0.17; 0.32]	0.14	0.52	0.606	0.000
Year 2	-0.18	[-0.32; -0.05]	0.07	-2.81	0.005**	0.008	-0.22	[-0.37; -0.07]	0.07	-3.08	0.002**	0.009
Year 3	-0.29	[-0.44; -0.15]	0.08	-3.73	<.001***	0.008	-0.32	[-0.49; -0.16]	0.09	-3.72	<.001***	0.009
Gender	-0.34	[-0.52; -0.16]	0.11	-3.11	0.002**	0.006	-0.33	[-0.53; -0.13]	0.12	-2.79	0.005**	0.006
Social-class × Year 2	0.03	[-0.10; 0.17]	0.07	0.51	0.612	0.000	0.06	[-0.08; 0.21]	0.07	0.90	0.369	0.001
Social-class × Year 3	-0.03	[-0.17; 0.12]	0.08	-0.34	0.734	0.000	-0.03	[-0.20; 0.13]	0.09	-0.37	0.713	0.001
Social-class × Gender	-0.09	[-0.27; 0.09]	0.11	-0.81	0.419	0.000	-0.13	[-0.33; 0.07]	0.12	-1.10	0.273	0.001
Intercept	6.32	[6.26; 6.38]	0.03	212.76	<.001***		6.30	[6.23; 6.36]	0.03	190.45	<.001***	
Social-class	-0.06	[-0.12; 0.00]	0.03	-2.00	0.045	0.002	-0.05	[-0.12; 0.01]	0.03	-1.62	0.105	0.001
Missing on-campus class												
Intercept	6.51	[6.19; 6.82]	0.16	39.98	<.001***		6.47	[6.12; 6.82]	0.18	36.07	<.001***	
Social-class	0.04	[-0.05; 0.12]	0.04	0.88	0.379	0.000	0.08	[-0.01; 0.17]	0.05	1.65	0.100	0.001
Year 2	-0.54	[-0.73; -0.35]	0.10	-5.62	<.001***	0.016	-0.59	[-0.80; -0.37]	0.11	-5.41	<.001***	0.016
Year 3	-0.46	[-0.67; -0.25]	0.11	-4.40	<.001***	0.016	-0.39	[-0.62; -0.16]	0.12	-3.40	0.001	0.016
Gender	-0.31	[-0.57; -0.06]	0.14	-2.27	0.023	0.003	-0.31	[-0.59; -0.02]	0.15	-2.03	0.043	0.003
Intercept	6.46	[6.15; 6.78]	0.16	40.27	<.001***		6.47	[6.12; 6.82]	0.18	36.06	<.001***	
Social-class	-0.27	[-0.59; 0.04]	0.16	-1.70	0.090	0.000	-0.12	[-0.47; 0.23]	0.18	-0.67	0.500	0.000
Year 2	-0.54	[-0.73; -0.35]	0.10	-5.56	<.001***	0.016	-0.58	[-0.79; -0.37]	0.11	-5.37	<.001***	0.016
Year 3	-0.47	[-0.68; -0.26]	0.11	-4.38	<.001***	0.016	-0.39	[-0.62; -0.15]	0.12	-3.36	0.001**	0.016
Gender	-0.27	[-0.53; -0.01]	0.14	-2.00	0.046	0.002	-0.31	[-0.59; -0.02]	0.15	-2.02	0.043	0.002
Social-class × Year 2	0.00	[-0.19; 0.20]	0.10	0.04	0.968	0.000	0.15	[-0.07; 0.36]	0.11	1.36	0.173	0.001

	Social-class$_{Education}$						Social-class$_{Occupation}$					
Variables	Estimate	[95% CI]	*SE*	*t*	*p*	η^2_p	Estimate	[95% CI]	*SE*	*t*	*p*	η^2_p
Social-class × Year 3	0.05	[-0.16; 0.27]	0.11	0.50	0.616	0.000	0.15	[-0.08; 0.39]	0.12	1.34	0.180	0.001
Social-class × Gender	0.26	[0.00; 0.52]	0.14	1.93	0.053	0.002	0.09	[-0.19; 0.38]	0.15	0.61	0.539	0.000
Intercept	5.84	[5.76; 5.93]	0.04	137.11	<.001***		5.81	[5.72; 5.91]	0.05	123.88	<.001***	
Social-class	0.05	[-0.04; 0.13]	0.04	1.12	0.261	0.001	0.08	[-0.01; 0.17]	0.05	1.75	0.080	0.002
**7. Out-of-class behaviors**												
Individual homework												
Intercept	5.28	[4.99; 5.58]	0.15	34.69	<.001***		5.25	[4.93; 5.57]	0.17	30.75	<.001***	
Social-class	0.05	[-0.03; 0.13]	0.04	1.23	0.220	0.001	0.05	[-0.04; 0.13]	0.04	1.08	0.278	0.001
Year 2	-0.07	[-0.25; 0.10]	0.09	-0.82	0.414	0.001	-0.09	[-0.28; 0.11]	0.10	-0.86	0.389	0.001
Year 3	0.05	[-0.15; 0.24]	0.10	0.48	0.634	0.001	0.06	[-0.15; 0.28]	0.11	0.59	0.558	0.001
Gender	-0.41	[-0.65; -0.17]	0.12	-3.30	0.001**	0.005	-0.36	[-0.62; -0.10]	0.14	-2.59	0.010	0.004
Intercept	5.26	[4.96; 5.55]	0.15	34.01	<.001***		5.24	[4.92; 5.57]	0.17	30.60	<.001***	
Social-class	-0.17	[-0.47; 0.12]	0.15	-1.11	0.266	0.000	-0.17	[-0.49; 0.16]	0.17	-0.98	0.329	0.000
Year 2	-0.08	[-0.26; 0.11]	0.09	-0.81	0.421	0.001	-0.09	[-0.29; 0.11]	0.10	-0.90	0.369	0.001
Year 3	0.05	[-0.15; 0.25]	0.10	0.52	0.601	0.001	0.07	[-0.14; 0.29]	0.11	0.66	0.506	0.001
Gender	-0.38	[-0.63; -0.14]	0.13	-3.05	0.002**	0.004	-0.36	[-0.62; -0.09]	0.14	-2.53	0.012	0.004
Social-class × Year 2	0.01	[-0.17; 0.19]	0.09	0.15	0.878	0.000	0.02	[-0.17; 0.22]	0.10	0.22	0.823	0.002
Social-class × Year 3	0.09	[-0.11; 0.29]	0.10	0.88	0.378	0.000	0.21	[0.00; 0.43]	0.11	1.93	0.054	0.002
Social-class × Gender	0.17	[-0.07; 0.42]	0.13	1.37	0.170	0.001	0.14	[-0.13; 0.40]	0.14	0.96	0.338	0.001
Intercept	4.81	[4.74; 4.89]	0.04	120.52	<.001***		4.83	[4.75; 4.92]	0.04	111.56	<.001***	
Social-class	0.05	[-0.03; 0.13]	0.04	1.29	0.197	0.001	0.05	[-0.04; 0.13]	0.04	1.14	0.256	0.001
Group homework												
Intercept	2.91	[2.61; 3.22]	0.15	19.91	<.001***		2.85	[2.52; 3.19]	0.16	17.80	<.001***	

	Social-class$_{Education}$						Social-class$_{Occupation}$					
Variables	Estimate	[95% CI]	*SE*	*t*	*p*	η^2_p	Estimate	[95% CI]	*SE*	*t*	*p*	η^2_p
Social-class	-0.03	[-0.11; 0.05]	0.04	-0.83	0.409	0.000	0.02	[-0.07; 0.10]	0.04	0.35	0.727	0.000
Year 2	0.40	[0.22; 0.58]	0.09	4.32	<.001***	0.011	0.42	[0.22; 0.62]	0.10	4.15	<.001***	0.013
Year 3	0.41	[0.20; 0.61]	0.11	3.85	<.001***	0.011	0.46	[0.24; 0.68]	0.12	3.95	<.001***	0.013
Gender	-0.18	[-0.43; 0.07]	0.12	-1.53	0.127	0.001	-0.14	[-0.41; 0.13]	0.13	-1.09	0.278	0.001
Intercept	2.87	[2.57; 3.18]	0.15	19.31	<.001***		2.85	[2.51; 3.18]	0.16	17.65	<.001***	
Social-class	-0.29	[-0.59; 0.02]	0.15	-1.92	0.055	0.000	-0.10	[-0.43; 0.24]	0.16	-0.60	0.550	0.000
Year 2	0.40	[0.22; 0.59]	0.09	4.27	<.001***	0.010	0.42	[0.21; 0.62]	0.10	4.11	<.001***	0.012
Year 3	0.39	[0.19; 0.60]	0.11	3.68	<.001***	0.010	0.46	[0.24; 0.68]	0.12	3.90	<.001***	0.012
Gender	-0.14	[-0.39; 0.11]	0.12	-1.15	0.250	0.001	-0.13	[-0.40; 0.14]	0.13	-1.00	0.316	0.000
Social-class × Year 2	0.01	[-0.17; 0.20]	0.09	0.15	0.884	0.000	0.01	[-0.19; 0.22]	0.10	0.13	0.895	0.000
Social-class × Year 3	-0.03	[-0.23; 0.18]	0.11	-0.24	0.814	0.000	0.00	[-0.22; 0.23]	0.12	0.04	0.968	0.000
Social-class × Gender	0.23	[-0.02; 0.48]	0.12	1.87	0.061	0.001	0.09	[-0.18; 0.37]	0.13	0.74	0.461	0.000
Intercept	2.96	[2.88; 3.04]	0.04	71.90	<.001***		2.97	[2.88; 3.06]	0.04	66.11	<.001***	
Social-class	-0.04	[-0.12; 0.04]	0.04	-0.99	0.323	0.000	0.01	[-0.07; 0.10]	0.04	0.30	0.767	0.000
Looking over class notes												
Intercept	3.66	[3.38; 3.93]	0.14	26.11	<.001***		3.68	[3.37; 3.98]	0.16	23.56	<.001***	
Social-class	-0.05	[-0.12; 0.02]	0.04	-1.31	0.191	0.001	-0.02	[-0.10; 0.06]	0.04	-0.50	0.618	0.000
Year 2	-0.18	[-0.35; -0.01]	0.09	-2.14	0.032	0.014	-0.16	[-0.34; 0.03]	0.09	-1.70	0.090	0.009
Year 3	-0.52	[-0.70; -0.33]	0.10	-5.35	<.001***	0.014	-0.43	[-0.63; -0.22]	0.11	-4.04	<.001***	0.009
Gender	-0.10	[-0.33; 0.12]	0.11	-0.91	0.365	0.000	-0.13	[-0.38; 0.12]	0.13	-1.02	0.306	0.001
Intercept	3.63	[3.35; 3.91]	0.14	25.28	<.001***		3.66	[3.35; 3.96]	0.16	23.29	<.001***	
Social-class	-0.18	[-0.46; 0.10]	0.14	-1.25	0.210	0.000	-0.22	[-0.52; 0.09]	0.16	-1.38	0.167	0.000
Year 2	-0.20	[-0.37; -0.02]	0.09	-2.26	0.024	0.014	-0.17	[-0.35; 0.02]	0.09	-1.80	0.073	0.010
Year 3	-0.54	[-0.72; -0.35]	0.10	-5.48	<.001***	0.014	-0.44	[-0.64; -0.23]	0.11	-4.12	<.001***	0.010

	Social-class$_{Education}$						Social-class$_{Occupation}$					
Variables	Estimate	[95% CI]	*SE*	*t*	*p*	η^2_p	Estimate	[95% CI]	*SE*	*t*	*p*	η^2_p
Gender	-0.07	[-0.30; 0.16]	0.12	-0.60	0.552	0.000	-0.10	[-0.35; 0.15]	0.13	-0.81	0.416	0.000
Social-class × Year 2	-0.09	[-0.26; 0.08]	0.09	-1.03	0.305	0.001	-0.06	[-0.25; 0.13]	0.09	-0.65	0.517	0.000
Social-class × Year 3	-0.10	[-0.29; 0.09]	0.10	-1.05	0.293	0.001	-0.07	[-0.28; 0.13]	0.11	-0.70	0.485	0.000
Social-class × Gender	0.17	[-0.06; 0.40]	0.12	1.43	0.153	0.001	0.21	[-0.04; 0.46]	0.13	1.67	0.096	0.002
Intercept	3.34	[3.27; 3.42]	0.04	88.34	<.001***		3.37	[3.29; 3.45]	0.04	82.16	<.001***	
Social-class	-0.05	[-0.12; 0.03]	0.04	-1.20	0.231	0.001	-0.02	[-0.10; 0.06]	0.04	-0.43	0.668	0.000
Keeping up on readings												
Intercept	3.21	[2.93; 3.49]	0.14	22.31	<.001***		3.26	[2.95; 3.57]	0.16	20.57	<.001***	
Social-class	0.01	[-0.07; 0.08]	0.04	0.20	0.844	0.000	0.01	[-0.08; 0.09]	0.04	0.15	0.877	0.000
Year 2	-0.10	[-0.27; 0.07]	0.09	-1.12	0.263	0.001	-0.06	[-0.25; 0.13]	0.10	-0.65	0.517	0.000
Year 3	-0.08	[-0.27; 0.11]	0.10	-0.81	0.419	0.001	-0.06	[-0.27; 0.14]	0.11	-0.58	0.561	0.000
Gender	0.01	[-0.22; 0.24]	0.12	0.10	0.922	0.000	-0.04	[-0.29; 0.22]	0.13	-0.29	0.775	0.000
Intercept	3.17	[2.88; 3.45]	0.15	21.46	<.001***		3.25	[2.93; 3.56]	0.16	20.24	<.001***	
Social-class	-0.23	[-0.52; 0.05]	0.15	-1.57	0.116	0.000	-0.23	[-0.54; 0.09]	0.16	-1.40	0.160	0.000
Year 2	-0.09	[-0.26; 0.09]	0.09	-0.99	0.320	0.001	-0.07	[-0.26; 0.12]	0.10	-0.70	0.481	0.000
Year 3	-0.09	[-0.28; 0.10]	0.10	-0.90	0.368	0.001	-0.07	[-0.28; 0.14]	0.11	-0.64	0.520	0.000
Gender	0.05	[-0.19; 0.28]	0.12	0.38	0.702	0.000	-0.02	[-0.27; 0.24]	0.13	-0.12	0.906	0.000
Social-class × Year 2	0.07	[-0.11; 0.24]	0.09	0.74	0.459	0.000	0.00	[-0.19; 0.19]	0.10	0.04	0.967	0.000
Social-class × Year 3	0.00	[-0.19; 0.19]	0.10	-0.03	0.975	0.000	-0.01	[-0.22; 0.20]	0.11	-0.10	0.918	0.000
Social-class × Gender	0.19	[-0.04; 0.43]	0.12	1.60	0.109	0.001	0.21	[-0.05; 0.46]	0.13	1.59	0.111	0.001
Intercept	3.16	[3.09; 3.24]	0.04	83.00	<.001***		3.18	[3.10; 3.27]	0.04	76.48	<.001***	
Social-class	0.01	[-0.07; 0.08]	0.04	0.24	0.808	0.000	0.01	[-0.07; 0.09]	0.04	0.17	0.864	0.000
**8. Independent and other in-class behaviors**												

	Social-class$_{Education}$						Social-class$_{Occupation}$					
Variables	Estimate	[95% CI]	*SE*	*t*	*p*	η^2_p	Estimate	[95% CI]	*SE*	*t*	*p*	η^2_p
Asking questions												
Intercept	2.79	[2.52; 3.06]	0.14	20.57	<.001***		2.84	[2.54; 3.14]	0.15	18.91	<.001***	
Social-class	0.10	[0.03; 0.17]	0.04	2.68	0.007**	0.003	0.11	[0.03; 0.19]	0.04	2.65	0.008**	0.004
Year 2	-0.03	[-0.20; 0.13]	0.08	-0.40	0.689	0.000	-0.10	[-0.29; 0.08]	0.09	-1.12	0.262	0.001
Year 3	0.00	[-0.18; 0.18]	0.09	0.02	0.986	0.000	-0.02	[-0.22; 0.18]	0.10	-0.20	0.843	0.001
Gender	0.02	[-0.20; 0.24]	0.11	0.19	0.849	0.000	0.02	[-0.22; 0.26]	0.12	0.16	0.870	0.000
Intercept	2.77	[2.50; 3.04]	0.14	20.05	<.001***		2.85	[2.55; 3.15]	0.15	18.70	<.001***	
Social-class	0.02	[-0.26; 0.29]	0.14	0.11	0.909	0.000	0.20	[-0.10; 0.50]	0.15	1.30	0.195	0.000
Year 2	-0.01	[-0.17; 0.16]	0.08	-0.08	0.939	0.000	-0.09	[-0.28; 0.09]	0.09	-0.98	0.327	0.001
Year 3	-0.01	[-0.20; 0.17]	0.09	-0.15	0.882	0.000	-0.03	[-0.23; 0.17]	0.10	-0.25	0.801	0.001
Gender	0.04	[-0.19; 0.26]	0.11	0.33	0.738	0.000	0.01	[-0.24; 0.25]	0.12	0.06	0.953	0.000
Social-class × Year 2	0.16	[-0.01; 0.32]	0.08	1.83	0.067	0.003	0.15	[-0.03; 0.34]	0.09	1.64	0.100	0.004
Social-class × Year 3	-0.08	[-0.26; 0.10]	0.09	-0.85	0.394	0.003	-0.12	[-0.32; 0.09]	0.10	-1.11	0.267	0.004
Social-class × Gender	0.04	[-0.18; 0.27]	0.11	0.38	0.705	0.000	-0.10	[-0.35; 0.14]	0.12	-0.83	0.404	0.000
Intercept	2.80	[2.73; 2.88]	0.04	76.36	<.001***		2.82	[2.74; 2.90]	0.04	69.80	<.001***	
Social-class	0.10	[0.03; 0.17]	0.04	2.70	0.007**	0.003	0.11	[0.03; 0.19]	0.04	2.66	0.008**	0.004
Participating in discussions												
Intercept	3.01	[2.73; 3.30]	0.14	20.91	<.001***		3.07	[2.75; 3.38]	0.16	19.24	<.001***	
Social-class	0.05	[-0.03; 0.12]	0.04	1.19	0.234	0.001	0.10	[0.02; 0.18]	0.04	2.43	0.015	0.003
Year 2	-0.08	[-0.25; 0.10]	0.09	-0.88	0.379	0.001	-0.18	[-0.37; 0.01]	0.10	-1.84	0.066	0.002
Year 3	-0.12	[-0.31; 0.07]	0.10	-1.21	0.228	0.001	-0.13	[-0.34; 0.08]	0.11	-1.20	0.230	0.002
Gender	0.07	[-0.16; 0.30]	0.12	0.60	0.546	0.000	0.06	[-0.20; 0.31]	0.13	0.45	0.656	0.000
Intercept	3.01	[2.72; 3.30]	0.15	20.49	<.001***		3.07	[2.76; 3.39]	0.16	19.10	<.001***	
Social-class	0.04	[-0.25; 0.33]	0.15	0.25	0.800	0.000	0.15	[-0.16; 0.47]	0.16	0.95	0.344	0.000

	Social-class$_{Education}$						Social-class$_{Occupation}$					
Variables	Estimate	[95% CI]	*SE*	*t*	*p*	η^2_p	Estimate	[95% CI]	*SE*	*t*	*p*	η^2_p
Year 2	-0.08	[-0.25; 0.10]	0.09	-0.84	0.401	0.001	-0.17	[-0.36; 0.02]	0.10	-1.77	0.077	0.002
Year 3	-0.12	[-0.32; 0.07]	0.10	-1.22	0.224	0.001	-0.13	[-0.34; 0.08]	0.11	-1.22	0.223	0.002
Gender	0.07	[-0.16; 0.31]	0.12	0.61	0.543	0.000	0.05	[-0.20; 0.31]	0.13	0.39	0.694	0.000
Social-class × Year 2	0.01	[-0.16; 0.19]	0.09	0.16	0.871	0.000	0.06	[-0.13; 0.26]	0.10	0.66	0.508	0.001
Social-class × Year 3	-0.01	[-0.21; 0.18]	0.10	-0.13	0.899	0.000	-0.06	[-0.27; 0.15]	0.11	-0.51	0.608	0.001
Social-class × Gender	0.01	[-0.23; 0.24]	0.12	0.05	0.958	0.000	-0.05	[-0.31; 0.20]	0.13	-0.40	0.687	0.000
Intercept	3.03	[2.96; 3.11]	0.04	78.41	<.001***		3.03	[2.95; 3.12]	0.04	72.08	<.001***	
Social-class	0.05	[-0.03; 0.12]	0.04	1.23	0.220	0.001	0.10	[0.02; 0.19]	0.04	2.45	0.015	0.003
Answering questions												
Intercept	3.48	[3.19; 3.76]	0.14	24.39	<.001***		3.53	[3.22; 3.84]	0.16	22.52	<.001***	
Social-class	0.10	[0.02; 0.17]	0.04	2.60	0.009**	0.003	0.13	[0.05; 0.21]	0.04	3.11	0.002**	0.005
Year 2	-0.15	[-0.32; 0.03]	0.09	-1.65	0.099	0.005	-0.22	[-0.41; -0.03]	0.10	-2.23	0.026	0.005
Year 3	-0.31	[-0.50; -0.12]	0.10	-3.20	0.001**	0.005	-0.30	[-0.51; -0.10]	0.11	-2.87	0.004**	0.005
Gender	0.03	[-0.20; 0.26]	0.12	0.28	0.783	0.000	0.00	[-0.25; 0.25]	0.13	0.01	0.991	0.000
Intercept	3.46	[3.18; 3.75]	0.15	23.88	<.001***		3.54	[3.23; 3.85]	0.16	22.35	<.001***	
Social-class	0.09	[-0.20; 0.38]	0.15	0.61	0.539	0.000	0.28	[-0.04; 0.59]	0.16	1.74	0.083	0.000
Year 2	-0.13	[-0.30; 0.05]	0.09	-1.45	0.147	0.005	-0.21	[-0.40; -0.02]	0.10	-2.16	0.031	0.005
Year 3	-0.33	[-0.52; -0.14]	0.10	-3.37	0.001**	0.005	-0.31	[-0.52; -0.10]	0.11	-2.90	0.004**	0.005
Gender	0.04	[-0.19; 0.28]	0.12	0.38	0.708	0.000	-0.01	[-0.26; 0.25]	0.13	-0.07	0.946	0.000
Social-class × Year 2	0.08	[-0.09; 0.26]	0.09	0.94	0.345	0.002	0.05	[-0.14; 0.24]	0.10	0.51	0.609	0.001
Social-class × Year 3	-0.13	[-0.32; 0.07]	0.10	-1.29	0.199	0.002	-0.12	[-0.33; 0.09]	0.11	-1.13	0.258	0.001
Social-class × Gender	0.01	[-0.22; 0.25]	0.12	0.09	0.926	0.000	-0.12	[-0.37; 0.14]	0.13	-0.91	0.363	0.000
Intercept	3.38	[3.31; 3.46]	0.04	88.26	<.001***		3.38	[3.30; 3.46]	0.04	80.72	<.001***	

	Social-class$_{Education}$						Social-class$_{Occupation}$					
Variables	Estimate	[95% CI]	*SE*	*t*	*p*	η^2_p	Estimate	[95% CI]	*SE*	*t*	*p*	η^2_p
Social-class	0.10	[0.03; 0.18]	0.04	2.66	0.008**	0.003	0.13	[0.05; 0.21]	0.04	3.15	0.002**	0.005
Switching camera on												
Intercept	2.20	[1.96; 2.44]	0.12	17.88	<.001***		2.28	[2.01; 2.54]	0.13	16.89	<.001***	
Social-class	0.13	[0.07; 0.20]	0.03	4.03	<.001***	0.008	0.19	[0.12; 0.26]	0.04	5.26	<.001***	0.015
Year 2	-0.17	[-0.32; -0.02]	0.07	-2.38	0.017	0.008	-0.22	[-0.38; -0.05]	0.08	-2.72	0.007**	0.011
Year 3	0.18	[0.02; 0.35]	0.09	2.10	0.036	0.008	0.20	[0.02; 0.38]	0.10	2.03	0.043	0.011
Gender	-0.10	[-0.30; 0.09]	0.10	-1.05	0.295	0.000	-0.13	[-0.35; 0.09]	0.11	-1.22	0.224	0.001
Intercept	2.21	[1.96; 2.45]	0.13	17.37	<.001***		2.27	[2.00; 2.54]	0.14	16.65	<.001***	
Social-class	0.23	[-0.01; 0.48]	0.13	1.85	0.065	0.000	0.16	[-0.11; 0.43]	0.14	1.19	0.235	0.000
Year 2	-0.19	[-0.34; -0.05]	0.07	-2.63	0.009**	0.008	-0.22	[-0.38; -0.06]	0.08	-2.75	0.006**	0.011
Year 3	0.16	[0.00; 0.33]	0.09	1.85	0.064	0.008	0.20	[0.02; 0.38]	0.10	2.00	0.045	0.011
Gender	-0.10	[-0.30; 0.10]	0.10	-0.94	0.348	0.000	-0.12	[-0.34; 0.10]	0.11	-1.13	0.257	0.001
Social-class × Year 2	-0.16	[-0.31; -0.02]	0.07	-2.23	0.026	0.003	-0.06	[-0.22; 0.11]	0.08	-0.72	0.471	0.000
Social-class × Year 3	-0.16	[-0.33; 0.00]	0.09	-1.86	0.063	0.003	0.01	[-0.17; 0.19]	0.10	0.06	0.948	0.000
Social-class × Gender	0.00	[-0.20; 0.20]	0.10	0.00	0.996	0.000	0.04	[-0.18; 0.26]	0.11	0.39	0.694	0.000
Intercept	2.07	[2.01; 2.14]	0.03	62.44	<.001***		2.11	[2.03; 2.18]	0.04	57.89	<.001***	
Social-class	0.14	[0.07; 0.20]	0.03	4.11	<.001***	0.008	0.19	[0.12; 0.26]	0.04	5.26	<.001***	0.015
Non-related activities												
Intercept	4.21	[3.94; 4.47]	0.14	30.26	<.001***		4.15	[3.86; 4.44]	0.15	27.30	<.001***	
Social-class	0.05	[-0.02; 0.12]	0.04	1.28	0.201	0.001	0.05	[-0.03; 0.12]	0.04	1.22	0.222	0.001
Year 2	0.08	[-0.08; 0.24]	0.08	0.94	0.350	0.012	0.07	[-0.11; 0.24]	0.09	0.73	0.466	0.010
Year 3	0.45	[0.27; 0.63]	0.09	5.08	<.001***	0.012	0.39	[0.20; 0.58]	0.10	4.01	<.001***	0.010
Gender	-0.03	[-0.24; 0.19]	0.12	-0.25	0.802	0.000	0.03	[-0.20; 0.27]	0.12	0.26	0.796	0.000

	Social-class$_{Education}$						Social-class$_{Occupation}$					
Variables	Estimate	[95% CI]	*SE*	*t*	*p*	η^2_p	Estimate	[95% CI]	*SE*	*t*	*p*	η^2_p
Intercept	4.18	[3.91; 4.45]	0.14	29.77	<.001***		4.15	[3.86; 4.44]	0.15	27.04	<.001***	
Social-class	-0.20	[-0.46; 0.07]	0.14	-1.41	0.158	0.000	-0.05	[-0.33; 0.24]	0.15	-0.30	0.767	0.000
Year 2	0.09	[-0.07; 0.25]	0.08	1.07	0.283	0.013	0.06	[-0.11; 0.24]	0.09	0.71	0.479	0.010
Year 3	0.46	[0.28; 0.64]	0.09	5.21	<.001***	0.013	0.40	[0.21; 0.59]	0.10	4.09	<.001***	0.010
Gender	-0.01	[-0.23; 0.21]	0.12	-0.10	0.923	0.000	0.03	[-0.20; 0.27]	0.13	0.26	0.798	0.000
Social-class × Year 2	0.10	[-0.06; 0.26]	0.08	1.21	0.228	0.001	0.01	[-0.17; 0.19]	0.09	0.11	0.914	0.001
Social-class × Year 3	0.15	[-0.02; 0.33]	0.09	1.73	0.084	0.001	0.14	[-0.05; 0.33]	0.10	1.46	0.144	0.001
Social-class × Gender	0.15	[-0.07; 0.37]	0.12	1.29	0.196	0.001	0.05	[-0.19; 0.28]	0.13	0.37	0.712	0.000
Intercept	4.32	[4.25; 4.39]	0.04	120.94	<.001***		4.31	[4.24; 4.39]	0.04	111.58	<.001***	
Social-class	0.04	[-0.03; 0.11]	0.04	1.23	0.219	0.001	0.05	[-0.03; 0.12]	0.04	1.18	0.240	0.001
Taking notes												
Intercept	6.45	[6.18; 6.71]	0.15	42.20	<.001***		6.42	[6.12; 6.72]	0.17	38.34	<.001***	
Social-class	0.01	[-0.06; 0.08]	0.04	0.32	0.746	0.000	-0.01	[-0.09; 0.07]	0.04	-0.26	0.796	0.000
Year 2	0.14	[-0.03; 0.30]	0.08	1.63	0.103	0.001	0.15	[-0.03; 0.33]	0.09	1.61	0.107	0.002
Year 3	0.02	[-0.16; 0.21]	0.09	0.26	0.793	0.001	0.02	[-0.18; 0.22]	0.10	0.23	0.818	0.002
Gender	-0.68	[-0.90; -0.46]	0.13	-5.19	<.001***	0.016	-0.65	[-0.90; -0.41]	0.14	-4.57	<.001***	0.015
Intercept	6.41	[6.14; 6.69]	0.15	41.67	<.001***		6.41	[6.11; 6.71]	0.17	38.02	<.001***	
Social-class	-0.19	[-0.46; 0.08]	0.15	-1.25	0.213	0.000	-0.10	[-0.40; 0.20]	0.17	-0.59	0.557	0.000
Year 2	0.13	[-0.04; 0.29]	0.08	1.53	0.126	0.001	0.14	[-0.04; 0.33]	0.09	1.54	0.123	0.001
Year 3	0.02	[-0.17; 0.20]	0.10	0.18	0.857	0.001	0.02	[-0.18; 0.22]	0.10	0.24	0.812	0.001
Gender	-0.64	[-0.87; -0.42]	0.13	-4.91	<.001***	0.014	-0.64	[-0.89; -0.40]	0.14	-4.46	<.001***	0.015
Social-class × Year 2	-0.03	[-0.20; 0.13]	0.08	-0.40	0.689	0.000	-0.05	[-0.23; 0.13]	0.09	-0.54	0.589	0.000
Social-class × Year 3	0.00	[-0.19; 0.18]	0.10	-0.03	0.976	0.000	0.05	[-0.15; 0.25]	0.10	0.45	0.651	0.000

	Social-class$_{Education}$						Social-class$_{Occupation}$					
Variables	Estimate	[95% CI]	*SE*	*t*	*p*	η^2_p	Estimate	[95% CI]	*SE*	*t*	*p*	η^2_p
Social-class × Gender	0.19	[-0.03; 0.42]	0.13	1.48	0.139	0.001	0.08	[-0.16; 0.33]	0.14	0.59	0.557	0.000
Intercept	5.75	[5.67; 5.82]	0.04	156.47	<.001***		5.75	[5.67; 5.83]	0.04	142.60	<.001***	
Social-class	0.01	[-0.06; 0.08]	0.04	0.31	0.757	0.000	-0.01	[-0.09; 0.07]	0.04	-0.17	0.861	0.000
Listening/reading carefully												
Intercept	5.30	[5.07; 5.52]	0.12	44.33	<.001***		5.25	[5.00; 5.50]	0.13	39.28	<.001***	
Social-class	-0.03	[-0.09; 0.03]	0.03	-1.11	0.265	0.001	-0.03	[-0.10; 0.03]	0.03	-1.02	0.306	0.001
Year 2	-0.09	[-0.23; 0.05]	0.07	-1.32	0.187	0.006	-0.04	[-0.19; 0.12]	0.08	-0.48	0.628	0.003
Year 3	-0.28	[-0.43; -0.13]	0.08	-3.57	<.001***	0.006	-0.19	[-0.36; -0.02]	0.09	-2.19	0.028	0.003
Gender	-0.21	[-0.39; -0.02]	0.10	-2.12	0.034	0.002	-0.20	[-0.40; 0.01]	0.11	-1.80	0.072	0.002
Intercept	5.27	[5.05; 5.50]	0.12	43.68	<.001***		5.24	[4.99; 5.50]	0.13	38.98	<.001***	
Social-class	-0.11	[-0.33; 0.12]	0.12	-0.88	0.377	0.000	-0.10	[-0.35; 0.16]	0.13	-0.72	0.473	0.000
Year 2	-0.08	[-0.22; 0.05]	0.07	-1.20	0.229	0.007	-0.04	[-0.19; 0.11]	0.08	-0.52	0.605	0.003
Year 3	-0.30	[-0.46; -0.15]	0.08	-3.84	<.001***	0.007	-0.20	[-0.37; -0.03]	0.09	-2.29	0.022	0.003
Gender	-0.18	[-0.37; 0.00]	0.10	-1.84	0.066	0.002	-0.18	[-0.39; 0.02]	0.11	-1.67	0.095	0.002
Social-class × Year 2	0.03	[-0.11; 0.16]	0.07	0.36	0.716	0.002	-0.02	[-0.17; 0.14]	0.08	-0.23	0.816	0.001
Social-class × Year 3	-0.14	[-0.29; 0.01]	0.08	-1.74	0.082	0.002	-0.11	[-0.27; 0.06]	0.09	-1.19	0.234	0.001
Social-class × Gender	0.09	[-0.10; 0.28]	0.10	0.90	0.371	0.000	0.09	[-0.12; 0.29]	0.11	0.78	0.434	0.000
Intercept	4.96	[4.90; 5.02]	0.03	162.10	<.001***		4.97	[4.90; 5.04]	0.03	146.55	<.001***	
Social-class	-0.03	[-0.09; 0.03]	0.03	-1.03	0.302	0.000	-0.03	[-0.10; 0.03]	0.03	-0.97	0.334	0.001

Note. Social-class (-1 = working-class; 1 = upper-middle-class); Year (reference category = first-year; 1 = second-year; 2 = third-year); gender (1 = other categories, 2 = male). Statistical differences robust to the Benjamini-Hochberg correction with $\alpha_{adjusted}$ = .01 are highlighted as follows: ***p* < .01; ****p* < .001. Degrees of freedom for model 1 (regressions on social-class, year, and gender) = 2165, for model 2 (regressions additionally on an interactive effect of social-class × year, and social-class × gender) = 2162, and model 3 (regressions on social-class) = 2168. [a] binomial logistic models with *z*-values and odds ratio with confidence interval. η^2_p = partial eta squared, *OR* = odds ratio, CI = confidence interval, IC = internet connection, HSI = high-speed internet, *f* = frequency.
